# Unique cellular protrusions mediate breast cancer cell migration by tethering to osteogenic cells

**DOI:** 10.1038/s41523-020-00183-8

**Published:** 2020-09-07

**Authors:** Aaron M. Muscarella, Wei Dai, Patrick G. Mitchell, Weijie Zhang, Hai Wang, Luyu Jia, Fabio Stossi, Michael A. Mancini, Wah Chiu, Xiang H.-F. Zhang

**Affiliations:** 1 Lester and Sue Smith Breast Center, Baylor College of Medicine, One Baylor Plaza, Houston, TX 77030 USA; 2 Dan L. Duncan Cancer Center, Baylor College of Medicine, One Baylor Plaza, Houston, TX 77030 USA; 3 Department of Molecular and Cellular Biology, Baylor College of Medicine, One Baylor Plaza, Houston, TX 77030 USA; 4 Graduate Program in Integrative Molecular and Biomedical Sciences, Baylor College of Medicine, One Baylor Plaza, Houston, TX 77030 USA; 5 Department of Cell Biology and Neuroscience, Institute for Quantitative Biomedicine, Rutgers University, 174 Frelinghuysen Road, Piscataway, NJ 08854 USA; 6 Department of Biochemistry and Molecular Biology, Baylor College of Medicine, One Baylor Plaza, Houston, TX 77030 USA; 7 National Center for Macromolecular Imaging, Baylor College of Medicine, One Baylor Plaza, Houston, TX 77030 USA; 8 Department of Bioengineering, Stanford University, Stanford, CA 94305 USA; 9 McNair Medical Institute, Baylor College of Medicine, One Baylor Plaza, Houston, TX 77030 USA

**Keywords:** Breast cancer, Cell migration, Cytoskeleton

## Abstract

Migration and invasion are key properties of metastatic cancer cells. These properties can be acquired through intrinsic reprogramming processes such as epithelial-mesenchymal transition. In this study, we discovered an alternative “migration-by-tethering” mechanism through which cancer cells gain the momentum to migrate by adhering to mesenchymal stem cells or osteoblasts. This tethering is mediated by both heterotypic adherens junctions and gap junctions, and leads to a unique cellular protrusion supported by cofilin-coated actin filaments. Inhibition of gap junctions or depletion of cofilin reduces migration-by-tethering. We observed evidence of these protrusions in bone segments harboring experimental and spontaneous bone metastasis in animal models. These data exemplify how cancer cells may acquire migratory ability without intrinsic reprogramming. Furthermore, given the important roles of osteogenic cells in early-stage bone colonization, our observations raise the possibility that migration-by-tethering may drive the relocation of disseminated tumor cells between different niches in the bone microenvironment.

## Introduction

Metastasis is the major challenge in research and treatment of cancers^1–3^. The metastasis cascade involves cellular migration. This is intuitively obvious as cancer cells are expected to leave primary tumors and enter distant organs, and almost every step of this process requires mobility. Multiple cell-intrinsic migratory mechanisms can be activated by epithelial-mesenchymal transition (EMT), during which cancer cells lose epithelial traits and gain motility^4^. More recently, a basal-like reprogramming process has been observed to render cancer cells migratory without losing cell-cell adhesions, also known as “collective migration”^5–7^. Both EMT and collective migration can be induced by the microenvironment through paracrine or direct cell-cell contact. In particular, a recent study demonstrated that cancer-associated fibroblasts (CAFs) can lead collective migration through development of heterotypic adherens junctions constituted by N-cadherin of CAFs and E-cadherin of cancer cells^8^. These findings provide novel insights into the escape of tumor cells from primary tumors.

After disseminated tumor cells (DTCs) enter distant organs such as bone, cellular migration may still be required before full colonization is accomplished. Increasing evidence suggests that the microenvironment in distant organs is compartmentalized into various niches. The unique cellular components and extracellular matrix of each niche determine the fate of cancer cells^9^. The perivascular niche, for instance, has been shown to maintain dormancy of DTCs^10,11^. On the other hand, we and others elucidated that the osteogenic niche promotes proliferation and early-stage bone colonization^12–14^. Thus, relocation between different microenvironment niches may represent one mechanism to switch cellular fates of DTCs. This relocation may involve a distinctive type of migration, which occurs when cancer cells exist as single- or few-cell micrometastases and are fully surrounded by various normal cells – a situation in contrast to what is known for migration and invasion in primary tumors. The cellular/molecular nature of this type of migration is completely elusive.

Here, we observed that proximity of cancer cells to osteogenic cells (e.g., mesenchymal stem cells and osteoblasts) increases the mobility of the former, and allows the otherwise inert cancer cells to move toward osteogenic signals. This process is mediated by a dendritic spine-like structure (DSLS) of cancer cells, which physically attaches to osteogenic cells through heterotypic adherens junctions and gap junctions. DSLS is supported by actin filaments coated with cofilin, and therefore, is highly pliable. Both gap junctions and cofilin are necessary for the maintenance of DSLS and for this “migration-by-tethering” mechanism. Evidence of DSLS in vivo was also obtained and suggests that the “migration-by-tethering” may represent a unique migration process of DTCs in the bone microenvironment.

## Results

### Cancer cells gain mobility through a MBT mechanism in co-culture with osteogenic cells

**Fig. 1:**
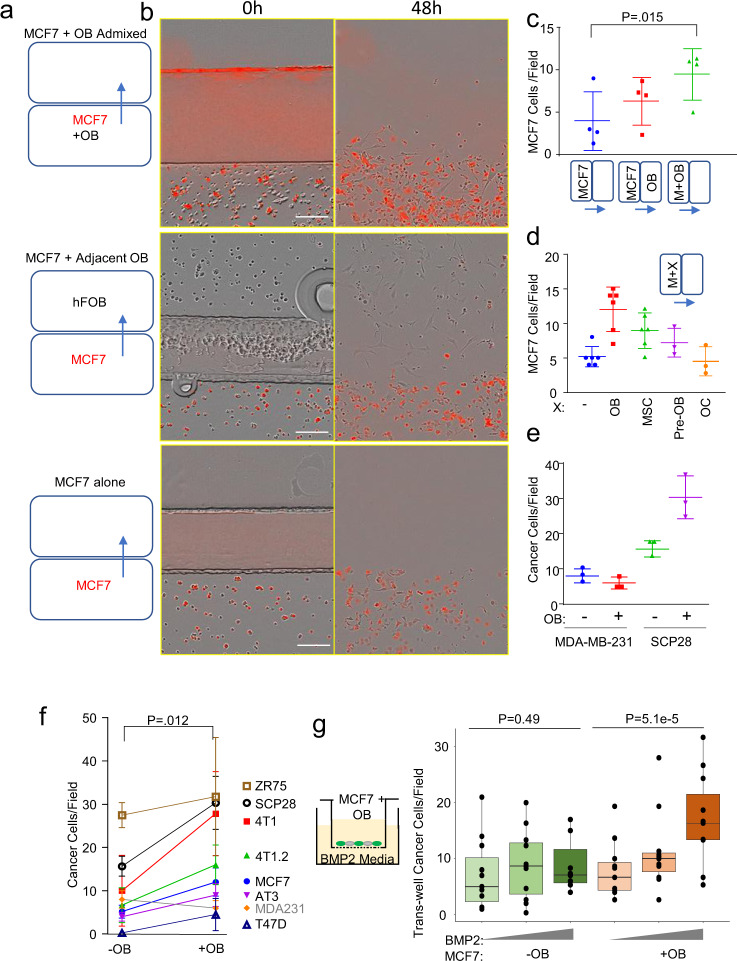
Breast cancer cells which express E-cadherin have increased migration when cultured with osteogenic cells in vitro. **a** Ibidi silicone removable chambers were used to quantify migration by breast cancer cells in monoculture, in co-culture with osteoblasts in adjacent chambers, or in direct admixture in the same chamber. **b** To distinguish cell types, fluorescently labeled breast cancer cells were co-cultured with unlabeled osteoblasts (OBs), and imaged with the Incucyte S3. Scale bar is 100 µm. **c** Dot plot showing the number of cells escaped from their original enclosure per field for MCF-7 alone, in co-culture with OBs in adjacent regions, or in co-culture with osteoblasts in the same region after 48 h. *P*-value determined by students *t*-test (two-tailed). Each dot represents a biological replicate, an average of three technical replicates. **d** Dot plot shows the number of cells escaped per field for MCF-7 alone, and in co-culture with OBs (represented by hFOB1.19 cells), pre-osteoblasts (Pre-OB represented by MC3T3-E1 cells), human mesenchymal stem cells (MSCs), and osteoclasts (OC RANK-L differentiated Raw 264.7 cells) after 48 h. Each dot represents a technical replicate. **d** Dot plot shows the number of cells escaped per field for mesenchymal MDA-MB-231 alone and with osteoblasts, as well as MDA-MB-231 E-cadherin expressing subline SCP-28 alone and with osteoblasts after 48 h. Each dot represents a technical replicate. **e** Dot plot shows the combined data for cells escaped per field for various E-cadherin expressing breast cancer cell lines alone and with osteoblasts. For contrast MDA-MB-231, which lacks E-cadherin expression, is also shown (not included in statistical comparison). *P*-value determined by students paired *t*-test (two-tailed). Each dot represents a biological replicate, an average of at least three technical replicates. **f** Box plot shows the transwell migration (a diagram shown in the left) of MCF-7 cells toward a gradient (0, 1, and 5 µM) of Bone Morphogenetic Protein-2 (BMP-2) with or without admixture of OBs. Each dot represents a biological replicate generated from an independent experiment average of three technical replicates. For **c**–**f** error bars indicate standard deviation. (For **g**), the lower and upper hinges correspond to the first and third quartiles (the 25th and 75th percentiles). The upper whisker extends from the hinge to the largest value no further than 1.5 × IQR from the hinge. The lower whisker extends from the hinge to the smallest value at most 1.5 × IQR of the hinge. Data beyond the end of the whiskers are called “outlying” points and are plotted individually. *P*-value as determined by analysis of covariance (ANCOVA) as elaborated in “Methods”.

Our previous studies have established a central role of osteogenic cells (the cells in the osteogenic lineage from MSCs to newly developed osteoblasts) in early-stage bone colonization^12,13^. These cells constitute the osteogenic niche and support cancer cell proliferation through adherens junctions and gap junctions. Cancer cells and osteogenic cells readily establish direct cell-cell contact in vivo and in 3D suspension co-cultures (Supplementary Fig. 1a), making us hypothesize chemotaxis between the two cell types. To test this hypothesis, we set up a simple two-chamber culture system as illustrated in Fig. 1a. The chamber walls can be removed to allow originally separated cells to interact or migrate towards the other chamber. We started with MCF-7 cells because these ER+ breast cancer cells can home to the osteogenic niche in vivo and form extensive cell-cell contact with osteogenic cells in 3D co-cultures as we showed in our previous works^12^.

Inconsistent to our hypothesis, MCF-7 cells did not exhibit chemotactic movement toward osteogenic cells in the adjacent chamber (Fig. 1b, c). In fact, MCF-7 cells maintain epithelial traits and are poorly migratory: when cultured alone, very few cells could move across the border between the chambers (Fig. 1b and Supplementary Video 1). However, when they were directly admixed with osteoblasts in the same chamber, the frequency of MCF-7 cells that could migrate across chambers evidently increased (Fig. 1b, c). Real-time imaging revealed that cancer cells stick to the much more mobile osteogenic cells and are “dragged” to move (Supplementary Fig. 1b and Supplementary Video 2), a phenomenon that we termed “migration-by-tethering” (MBT). We next asked if other major cell types in the bone microenvironment can also drive MBT. Human mesenchymal stem cells (hMSCs) and MC3T3-E1 pre-osteoblasts cells showed this ability when co-cultured with MCF-7. However, RANK-L differentiated Raw 264.7 osteoclasts failed to increase of MCF-7 motility (Fig. 1d). Thus, it suggests that, for cancer cells in the bone, MBT can be mediated by cells with osteogenic potential (i.e., osteogenic cells).

To examine cancer cell specificity related to MBT, we tested several more human and murine cancer cell lines known to establish bone metastases according to previous studies, namely 4T1, 4T1.2, AT3, and SCP28^13,14^. All of these lines exhibited similar MBT properties (Fig. 1e, f and Supplementary 1c). In particular, SCP28 is a single cell-derived, bone-seeking subpopulation of MDA-MB-231^15^. One difference between SCP28 and the parental population is the expression of E-cadherin (Supplementary Fig. 1d). Interestingly, migration of MDA-MB-231 parental cells did not increase when co-cultured with osteogenic cells (Fig. 1e), raising the possibility that E-cadherin is involved in MBT. In fact, other cell lines with MBT capacity are also E-cadherin positive as we previously showed (Fig. 1f)^12^. The potential role of E-cadherin in MBT will be further discussed in a later section.

In physiological conditions, osteogenic cells respond to osteogenic signals by chemotaxis and differentiation. Our initial observations suggest that cancer cells can be attracted to sources of osteogenic signals through MBT. We tested this possibility by transwell assays in which cancer cells and/or osteogenic cells are admixed in the upper chamber and BMP-2-containing media is placed in the bottom chamber. As expected, transwell migration of MCF-7 cells was changed very little by BMP-2 when they are cultured alone, but significantly increased when osteogenic cells are co-cultured (Fig. 1g and Supplementary Fig. 1e)—supporting that osteogenic cells and MBT may drive relocation of cancer cells toward sites releasing osteogenic signals. While direct treatment of BMP-2 can increase MCF-7 migration through transwell^16^, in this experiment, with cells at relatively low density and with BMP-2 used as a chemoattractant, no statistically significant difference was recorded. This increase is not due to proliferation, as proliferation inhibitors did not reduce the number of cells that cross the membrane when osteoblasts and BMP-2 are present (Supplementary Fig. 1f). Further experiments suggested that the increase in migration is osteoblast-contact-dependent, not a paracrine attraction, as osteoblasts in the bottom chamber of the transwell insert do not attract cancer cells on their own (Supplementary Fig. 1g). Additionally, we also excluded the possibility that this increase is due to extracellular matrix deposited by osteoblasts. When osteoblasts were removed with EDTA instead of trypsin, leaving osteoblast-produced ECM, the subsequently added cancer cells migrate notably less (Supplementary Fig. 1h), suggesting that live osteoblasts are the major driving force of the increased transwell migration toward osteogenic signals.

### Cancer cells adhere to osteogenic cells via DSLS

**Fig. 2:**
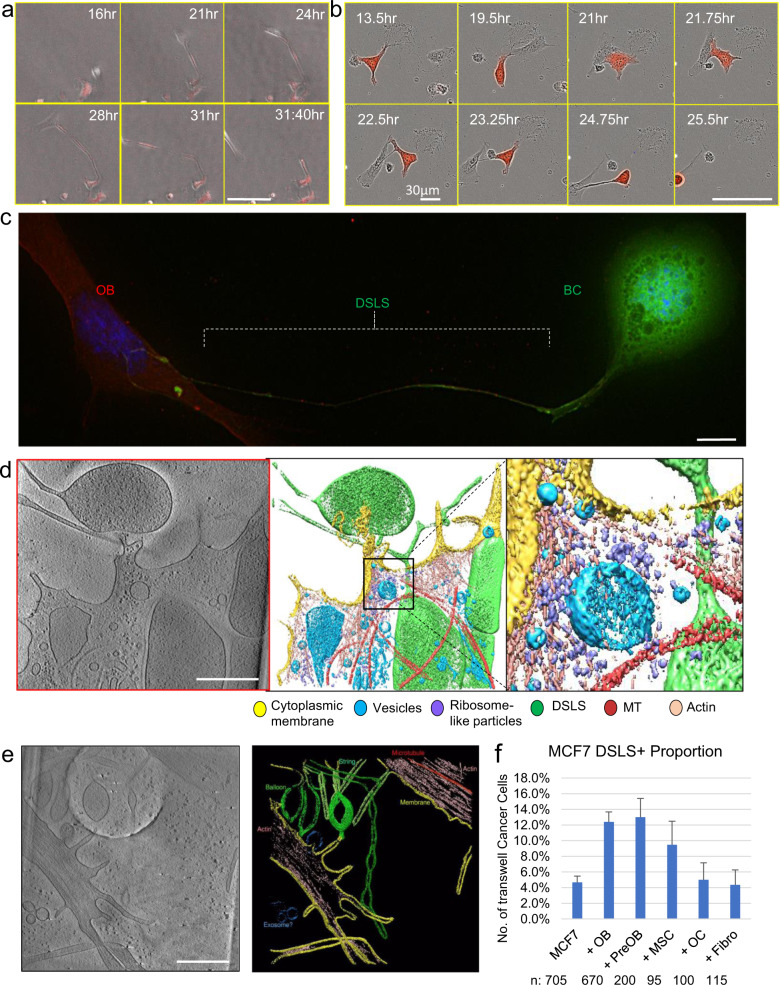
Luminal breast cancer cells form dendritic spine like protrusions that tether them to mobile osteoblasts. **a** Time-lapse on Cytation of mobile osteoblasts contacting and drawing out cellular protrusion from MCF-7 (red). Scale bar is 100 µm. **b** Time-lapse on Incucyte S3 of osteoblast (unlabeled) drawing out cellular tether protrusion that leads to MCF-7 (RFP-labeled,) migration. Scale bar is 100 µm. **c** Deconvolution imaging of MCF-7 cell (green) with dendritic spine-like structures (DSLS) protrusions attached to OBs (red). Scale bar is 10 µm. **d** Tomogram of the terminus of DSLS (green) touching OB cell membrane (yellow). Detail of actin and ribosome concentration where cells make contact. Scale bar is 500 nm. **e** Tomogram of multiple DSLS structures and actin within. Scale bar is 500 nm. **f** The relative abundance of MCF-7 DSLS structures in co-culture with various bone cells, normalized to abundance in monoculture. OB: osteoblasts represented by hFOB 1.19 cells. PreOB: Pre-osteoblasts represented by MC3T3-E1 cells. OC: osteoclasts represented by RAW 264.7 cells. MSC: immortalized Mesenchymal Stem cells Fibro: fibroblasts represented by 293T cells. Error bars indicate standard deviation calculated based on binomial distribution (see “Methods”).

We then set out to understand the cellular and molecular mechanisms underlying MBT. Higher-resolution, real-time microscopy revealed an interesting process: a long cellular protrusion was stretched out from cancer cells by osteoblasts that are migrating. This protrusion elongated as osteoblasts moved apart and maintained the direct cell-cell contact. In many cases, the protrusion was finally dissociated from osteogenic cells and regressed (Fig. 2a and Supplementary Video 3). In some cases, the connection eventually led to co-migration of cancer cells in an abrupt manner (Fig. 2b, Supplementary Videos 4 and 5). Through deconvolution microscopy, we observed that the protrusion structure morphologically resembles dendritic spines of neurons^17,18^—a small membranous protrusion from a neuron’s dendrite that typically receives input from a single axon at the synapse. It comprises of a thin neck that can be as long as 100 µm and usually with a spherical head at the terminal (Fig. 2c). Sometimes additional spherical structures can be found in the middle of the neck. Therefore, we named this protrusion dendritic spine-like structure, or DSLS.

We next used cryo-electron tomography (cryo-ET) to characterize DSLS at nm resolution, and confirmed that DSLS physically contacts osteogenic cells, and at that contact point there is an altered organization of the cytoskeleton (Fig. 2d). When cancer cells are not in proximity to osteogenic cells, shorter “buds” of DSLS could be observed at cell periphery, which are clearly distinct from typical filopodia, with regards to size and actin composition (filopodia contain actin bundles)^19^ (Fig. 2e). However, elongated DSLS increased when cancer cells were co-cultured with osteogenic cells, but not other bone cell types including fibroblasts and osteoclasts (Fig. 2f). We also observed DSLS in multiple other cancer cell lines including some non-breast cancers, suggesting that this is not a breast cancer-specific phenomenon (Supplementary Fig. 2a and Supplementary Table 1).

The morphological features distinguish DSLS from other previously described sub-cellular structures such as migrasomes^20^, tunneling nanotubes^21^ and cytonemes^22^. Migrasomes are derived from retraction fibers that trail behind migrating cells, and eventually are deposited behind the cells^20^. This is the opposite to DSLS, which leads the way of migration. We observed tunneling nanotubes between AT3 cells in their monocultures, usually in the form of multiple parallel tubes (Supplementary Fig. 2b). These tubes are not apparently associated with migration and morphologically distinct from DSLS connecting the cancer cells to osteoblasts. The expression of myosin X, a key component of cytonemes, did not change in co-cultures with osteoblasts (Supplementary Fig. 2c).

DSLS is also distinct from filopodia and lamellipodia, as indicated by its unique responses to actin/microtubules perturbations. While actin assembly inhibitor latrunculin B was shown to decrease filopodia formation^23^, this treatment increased DSLS development (Supplementary Fig. 2d). Jasplakinolide, an actin stabilizer and promotor of nucleation, was shown to decrease lamellipodia^24^, but again increased DSLS frequency (Supplementary Fig. 2d). The effect size of these two agents is ~3–5 fold by manual counting. Furthermore, nocodazole, a microtubule polymerization blocker, increasesd filopodia density^25^, but had no clear effect on DSLS prevalence or structure (Supplementary Fig. 2d). Taken together, DSLS appears to be distinctive from the previously characterized cellular protrusions.

### Cofilin is an essential cytoskeletal component of DSLS and necessary for MBT

**Fig. 3:**
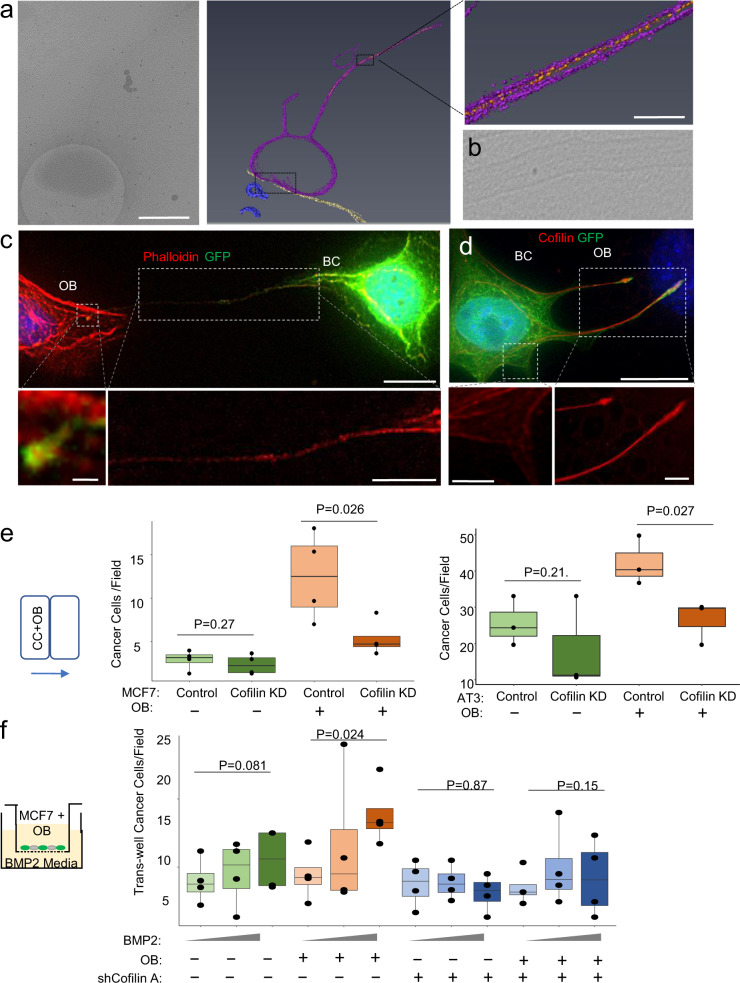
Protrusions are driven by actin, cofilin, adhere to OBs using E-Cadherin. **a** Cryo-ET tomogram showing actin fibers (orange) within the stalk of MCF-7 DSLS (purple) contacting an osteoblast (yellow). Scale bars are 500, 50 nm, respectively. **b** Micrograph from an additional MCF-7 DSLS showing actin fibers. **c** Phalloidin staining shows actin filaments in the DSLS stalk. Scale bars are 15 (upper), 1 (lower left), and 10 µm (lower right) respectively. **d** Cofilin immunolabeling of DSLS. The red channel intensity is matched for both the zoom of DSLS and of the cell body, normal filopodia to emphasize cofilin distribution. Scale bars are 15 (upper), 5 (lower left), and 5 µm (lower right), respectively. **e** Box and dot plots showing the cells per field migrated out of the chamber for MCF-7 and AT3 cells transfected with GIPZ shRNA construct Empty Vector (Control), and a shRNA against cofilin for knockdown (Cofilin KD), with and without OBs. Each dot represents a biological replicate, an average of three technical replicates. **f** Box plot showing the transwell migration cells through the membrane per field for MCF-7 transfected with GIPZ shRNA construct Empty Vector, and the shRNA against cofilin (Cofilin KD) with and without OBs. Each dot represents a biological replicate, an average of three technical replicates. For box plots, the lower and upper hinges correspond to the first and third quartiles (the 25th and 75th percentiles). The upper whisker extends from the hinge to the largest value no further than 1.5 × IQR from the hinge. The lower whisker extends from the hinge to the smallest value at most 1.5 × IQR of the hinge. Data beyond the end of the whiskers are called “outlying” points and are plotted individually. *P*-value as determined by either ANOVA (**e**) or ANCOVA (**f**) as elaborated in Methods.

We performed additional cryo-ET to visualize possible cytoskeleton supporting DSLS. Strand structures were observed in the neck of DSLS, suggesting actin filaments (Fig. 3a, b), which was confirmed by deconvolution microscopy and phalloidin staining (Fig. 3c). The pliability of DSLS led us to suspect that the actin filaments must be associated with other proteins that confer flexibility. Cofilin is a compelling candidate because it is known to sever actin filaments to allow bending^26–28^. Immunofluorescence staining confirmed this hypothesis. Indeed, cofilin was abundantly expressed along the long axis of DSLS and aligned well with actin filament (Fig. 3d). This distribution provides an explanation for the remarkable pliability of DSLS, and also distinguishes DSLS from regular filopodia in which cofilin is organized in parallel with membrane but perpendicular to direction of elongation (Fig. 3d, left inset). Expression of eGFP::cofilin fusion proteins confirmed that cofilin is present in the neck of DSLS, and enriched into small bands immediately adjacent to areas of increased flexibility of the structure (Supplementary Fig. 3a and Supplementary Video 6). The interaction between cancer cells and osteogenic cells modestly enhanced transcription of cofilin-encoding gene. (Supplementary Fig. 3b). To examine if cofilin is required for MBT, we knocked down (KD) cofilin by shRNAs in MCF-7 cells (Supplementary Fig. 3c). As expected, although cofilin KD did not affect MCF-7 migration in monocultures, it reduced the frequencies of prolonged DSLS (Supplementary Fig. 3d) and decreased co-migration of MCF-7, AT3, and 4T1 cells with osteogenic cells (Fig. 3e and Supplementary Fig. 3e). Consistently, chemotaxis of osteogenic cells toward BMP-2, the osteogenic signal, drives transwell migration of MCF-7 and 4T1 cells in a cofilin-dependent manner, supporting the MBT mechanism (Fig. 3f and Supplementary Fig. 3f). Of note, the degree of knockdown of cofilin did not seem sufficient to block migration of MCF-7 cells toward EGF (Supplementary Fig. 3g). Thus, the role of cofilin in MBT toward osteogenic signals appears to be more pronounced as compared to that in general migration. The phosphorylation status of cofilin is crucial to its function, with phosphorylated cofilin being the inactive form, with a variety of upstream mechanisms of regulation^29^. For this reason, we performed western blot and immunofluorescence staining of p-cofilin (Supplementary Fig. 3h, i). The signal was extremely weak in both assays, and appeared to be similar between monoculture vs coculture with osteoblasts. This may be consistent with p-cofilin being unbound to actin and dispersed in the cytoplasm^26^. Profilin, another small actin-binding protein involved in actin-polymerization dynamics, is prominently expressed by MCF-7 cells, but there is little change in profilin expression between monoculture and coculture, or when cofilin is knocked down (Supplementary Fig. 3j)^30^.

### Gap junctions between DSLS and osteogenic cells are necessary for DSLS maintenance and MBT

**Fig. 4:**
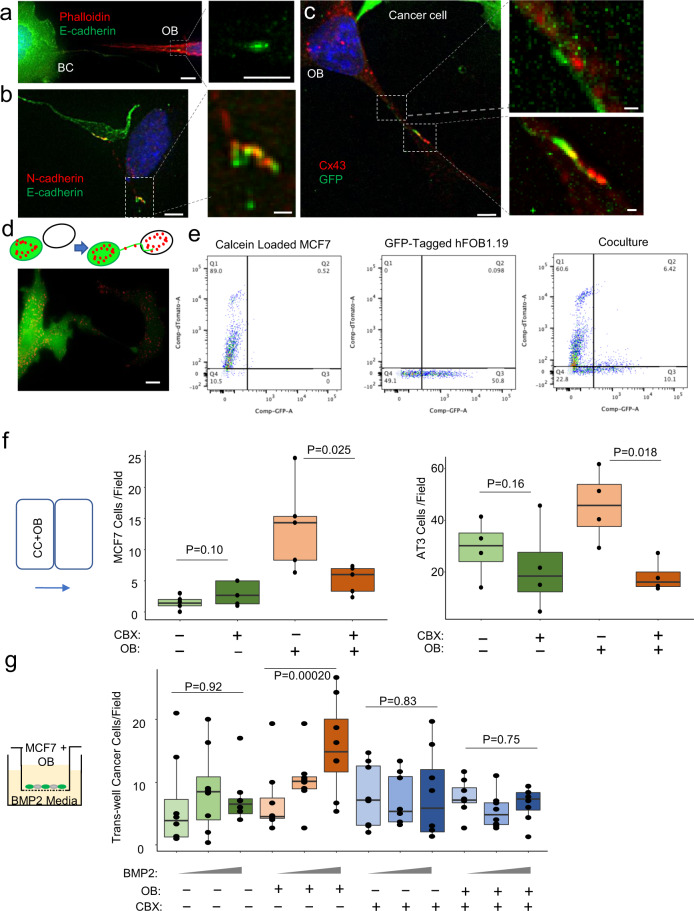
Protrusions facilitate gap junctions, calcium transfer between the cells. **a** MCF-7 E-cadherin (green) expressed where DSLS contacts osteoblast, here visible with phalloidin staining (red). Scale bars are 10 (left), and 5 µm (right), respectively. **b** MCF-7 E-cadherin (green) binding osteoblast N-cadherin (red) where DSLS contacts osteoblast. Scale bars are 5 (left), 1 µm (right), respectively. **c** Osteoblast gap junction protein connexin 43 (Cx43 – red) at the site of MCF-7 (green) DSLS contact. Scale bars are 5 (left), 1 (upper right), and 1 µm (lower right), respectively. **d** Gap junction permeable dye calcein red traversing from MCF-7 to osteoblast via DSLS. Scale bar is 15 µm. **e** Flow cytometry quantification of calcein dye transfer. The first panel shows un-transfected MCF-7 loaded with calcein red dye. The second shows GFP-labeled osteoblasts. The third shows the co-culture of the two cells after 18 h. **f** Box plots showing the 2D migration cells through the membrane per field for MCF-7 and AT3 cells treated with 10 µM CBX gap junction inhibitor with and without osteoblasts. Each dot represents a biological replicate, an average of three technical replicates. **g** Box plots showing the transwell migration cells through the membrane per field for MCF-7 cells treated with 10 µM CBX gap junction inhibitor with and without osteoblasts. Each dot represents a biological replicate, an average of three technical replicates. For box plots, the lower and upper hinges correspond to the first and third quartiles (the 25th and 75th percentiles). The upper whisker extends from the hinge to the largest value no further than 1.5 × IQR from the hinge. The lower whisker extends from the hinge to the smallest value at most 1.5 × IQR of the hinge. Data beyond the end of the whiskers are called “outlying” points and are plotted individually. *P*-value as determined by either ANOVA (**f**) or ANCOVA (**g**) as elaborated in “Methods”.

Bone micrometastases develop heterotypic adherens junctions (hAJs) and gap junctions (GJs) with osteogenic cells when they are in full contact^12,13^. Here, we asked whether the physical contact between DSLS and osteogenic cells is also mediated via hAJs and GJs, before more extensive cancer-osteogenic cell contact is established. Immunofluorescence staining revealed that E-cadherin is often concentrated at the head of DSLS (Fig. 4a), and co-localizes with N-cadherin (Fig. 4b).

Adherens junctions often precede the development of, and can stabilize GJs, which allow direct substance diffusion between the contacting cells^31–33^. Connexin 43 (Cx43), a major component of GJ in bone micrometastases^13^, was expressed at the interface between DSLS and osteogenic cells (Fig. 4c). Pre-incubation of cancer cells with calcein, a small fluorescence dye that can diffuse through GJ, clearly indicated GJ functions between cancer cells and osteogenic cells as shown by live cell fluorescence imaging and flow cytometry (Fig. 4d, e). We asked if GJs are necessary for DSLS development and MBT. Inhibition of GJ by an inhibitor, carbenoxolone (CBX), significantly decreased DSLS (Supplementary Fig. 4a) and reduced MBT in the 2D chamber and osteogenic transwell assay (Fig. 4f, g and Supplementary Fig. 4b). Similarly, a Cx43-specific inhibitory peptide, Gap19, achieved comparable effects and specifically block MBT in 2D and transwell settings (Supplementary Fig. 4c). Notably, the reduction of MBT is not due to the decrease of osteoblast migration (Supplementary Fig. 4d). Again, to address the specificity of BMP-2 and to distinguish MBT from cancer cell-intrinsic migration mechanisms, we also performed a similar experiment using EGF to replace BMP2 and found no significant reduction of EGF-induced cell migration from CBX (Supplementary Fig. 4e). To specifically disrupt Cx43 on either cell type, we transduced cancer cells with a dominant-negative mutant of Cx43, and transfected osteoblasts with Cx43-targeting siRNA before co-culture, respectively. Both treatments significantly decreased BMP-2-induced MBT (Supplementary Fig. 4f). Additionally, cofilin knockdown alone did not affect Cx43-expression by the cancer cells, indicating that Cx43 was independently important and not a downstream effect of cofilin loss (Supplementary Fig. 4g). These results support that the previously observed differences are indeed caused by inhibition of MBT but not general motility of cancer cells.

### In vivo evidence supporting the existence of DSLS in bone metastases

We asked if DSLS can be observed in bone lesions. Several difficulties were encountered. First, the morphologically distinct neck of DSLS in 3D tissues may be too long and thin to be completely visualized by a 2D focal plane, even when imaging a large field of view by microscopy. Second, MBT is transient. Hence, the time window to visualize this process may be narrow, requiring a large number of incidents to be monitored. Third, bone is a calcified tissue and highly auto-fluorescent, posing challenges to direct imaging. On the other hand, the lengthy decalcification process disrupted prolonged DSLS. These barriers hampered high-resolution imaging. Indeed, using regular approaches we could only detect very short DSLS-like structures in DTCs in the bone marrow (Supplementary Fig. 5a).

**Fig. 5:**
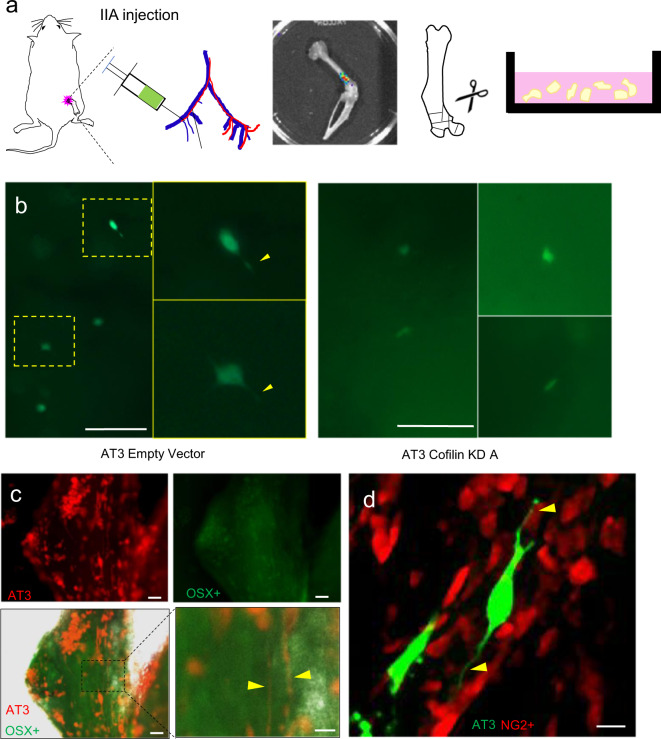
Evidence of DSLS in breast cancer bone metastasis. Labeled AT3 cells were injected via intra-iliac artery (IIA) injection into C57B/6 mice. Mice were immediately sacrificed, hind limb bones harvested, and cut into small (1–2 mm diameter) pieces. Bone fragments are cultured over the next 3 days and imaged each day. **a** AT3 cells with both empty vector and cofilin knockdown (KD) shRNA transfection in the bone 24 h after intra-iliac artery injection, sacrifice, and bone culture. Cofilin knockdown decreased the proportion of DTCs with visible DSLS from 50% to 16.7% (*n* = 9 mice). Yellow arrows indicate potential DSLS. Scale bars are 150 µm. **b** Spontaneous bone metastasis generated by orthotopic mammary tumors of RFP-tagged AT3. Hosts were OSX-GFP mouse mice. Red, green and merged channels are shown separately. Yellow arrows indicate potential DSLS in the vicinity of OSX+ cells. Scale bars are 50 (upper left), 50 (upper right), 50 (lower left), and 20 µm (lower right). **c** NG2-RFP mouse bone fragment containing GFP-tagged AT3 cells delivered by intra-iliac artery injection. Yellow arrows indicate DSLS from RFP-tagged AT3 cells terminating in contact with NG2+ mesenchymal stem cells. Scale bar is 15 µm.

We experimented several procedures to overcome the abovementioned difficulties. A bone-in-culture array (BICA)-based pipeline successfully revealed morphological features of cancer cells that colonize bones. Specifically, mouse hind limb bones containing either experimental bone lesions introduced via Intra-Iliac Artery Injection (IIA Inj.)^34^ or spontaneous bone metastases were extracted and fragmented to generate ex vivo bone segments, which were then subjected to light microscopy and deconvolution microscopy in live tissues (Fig. 5a). This approach was previously utilized to screen for drugs that selectively target cancer–bone interactions^35^. Using MCF-7 cells and IIA injection, we could detect GFP-tagged microscopic bone lesions as well as cellular protrusions that are morphological similar to DSLS in cultures (Supplementary Fig. 5b, c). Cofilin knockdown appeared to decrease such protrusions in both MCF-7 (Supplementary Fig. 5d) and AT3 within the bone (Fig. 5b). Using the murine AT3 line, spontaneous bone metastases comprising a larger number of cancer cells also exhibited prolonged cellular protrusions that are likely to represent DSLS (Fig. 5c). To ask whether the suspected DSLS mediated interactions between cancer cells and osteogenic cells, we examined spontaneous bone metastasis derived from RFP^+^ AT3 cells in osterix (OSX)-GFP mice in which osterix-expressing pre-osteoblast and osteoblast cells are GFP^+^. Under the BICA setting, we observed that cancer cells tend to localize to OSX-enriched regions and develop long cellular protrusions (Fig. 5c). In an independent experiment, GFP^+^ AT3 were injected with IIA into NG2-RFP mice. NG2 is a well-established marker of perivascular mesenchymal cells, and often used to indicate mesenchymal stem cells^36,37^. Confocal imaging showed that AT3 DTCs develop cellular protrusions that often terminate on NG2^+^ mesenchymal stem cells, supporting the hypothesis that DTCs can form DSLS with osteogenic cells in the bone (Fig. 5d).

## Discussion

Our cell biology experiments strongly suggest that DSLS mediates MBT with osteogenic cells, and may drive migration of cancer cells that do not possess intrinsic migratory properties. This led us to hypothesize that physiological and pathological mobilization of osteogenic cells may provide momentum for DTC redistribution in the bone microenvironment. For example, bone fractures activate chemotaxis of MSCs toward the injured site and stimulate subsequent osteogenic differentiation^38^. This process may coincide with migration of DTCs from the perivascular niche, where DTCs are kept dormant, to the osteogenic niche, where DTCs become proliferative. Consistent with this hypothesis, osteogenic MSCs have been reported to be perivascular in the resting stage^37,39^, and therefore may provide a transition between the two different types of niches and an exit of the dormancy state. Significant future work will be needed to test this hypothesis and validate the functionality of DSLS in vivo. Given numerous gap junction-inhibitors and the discovery of cofilin-binding oligosaccharide JG6, itself shown to be capable of suppressing lung metastasis in mice, there may be a future translation of this data to the prevention or suppression of bone metastasis^40,41^.

We realize that morphological evidence may not be sufficient to support the uniqueness of DSLS – it might indeed be molecularly related to one of these previously described structures. For instance, tunneling nanotubes (TNTs) have also been observed on MCF-7 cells in other studies^42–44^. In fact, actin-based TNTs expressed by macrophages have been shown to contact breast cancer cells and confer upon them increased invasive properties, and TNT-deficient macrophages led to decreased xenograft tumor growth in vivo^45,46^. However, there are significant differences in morphology between TNTs and DSLS in the same cell line (Supplementary Fig. 2b). In addition, at the functional level a cellular protrusion that mediates heterotypic cell-cell interaction and leads to co-migration between different cell types has not been described. Furthermore, the formation of such protrusions via initial contact and withdrawal, as well as the role of cofilin, is different than TNT formation described previously. In any case, it may be that DSLS and TNTs are closely related, but DSLS in context represent a new type of small actin-based protrusion function.

## Methods

### Reagents and antibodies

General laboratory reagents and chemicals were obtained from Thermo Fisher Scientific or Sigma-Aldrich unless specifically indicated. Inhibitor stocks were prepared in water as follows: 50 mM carbenoxolone (CBX) (Sigma-Aldrich), 2 mg/mL Gap19 (Tocris Bioscience), 2 mM Latrunculin B (Sigma-Aldrich), 100 nm Jasplakinolide (Sigma-Aldrich) and 250 nM Nocodazole (Sigma-Aldrich). Commercially available monoclonal antibodies (mAb) or polyclonal antibodies (pAb) were used for Cofilin (rabbit pAb, Abcam ab42824), GFP (chicken pAb, Abcam ab13970), E-cadherin (rabbit mAb, Cell Signaling Technologies 3195S), N-Cadherin (mouse mAb, Abcam ab98952, Notch3 (rabbit pAb, Abcam ab23426), Cx43 (rabbit pAb, Sigma C6219), Myosin X (rabbit pAb, Novus, 22430002)) and ALP (rabbit mAb, Abcam ab108337). To stain for actin Texas Red™-X Phalloidin (Thermo-Fisher T7471) was used. Dye was diluted (1:40) in 100% ethanol and exposed to fixed cells for 20 min at room temperature. Secondary goat antibodies against chicken, mouse, or rabbit labeled with Alexa Fluor 488, 594 or Cy5 were purchased from Jackson ImmunoResearch Laboratories Inc.

Red CellTrace™ Calcein AM Red-Orange was used to the track the formation of functional gap junctions between two cells. Dye was loaded by reconstituting 50 µg in 31 µL of anhydrous DMSO to make a 2 mM Calcein AM Stock Solution. Immediately prior to use, the Calcein AM Stock Solution was diluted in PBS to 2x Calcein AM Working Solution. Working solution was added to cells in suspension in PBS at a 1:1 ratio. The cells were then incubated for 30 min at 37 °C under 5% CO2, then washed with PBS 4 times before seeding.

### Molecular biology

pEGFP-N1 human cofilin WT was obtained from Addgene (Plasmid #50859) and cloned into pLJM1-EGFP (Plasmid #19319) by removing eGFP::Cofilin with Xba1 and Nhe1 and cloning into pLJM1-EGFP. EGFP was removed from pLJM1 with the same enzymes. Wild-type Cx43 and dominant negative Cx43 G138R was cloned from hFOB1.19 cDNA, mutations introduced by PCR. pInducer22 was used to express these genes in a doxycycline-induced overexpression system. To induce gene expression in vitro cells were treated with 50–100 ng/ml doxycycline in culture media.

GIPZ shRNA knockdown constructs were obtained from the BCM Cell-Based Assay Screening Core, and are originally from Dharmacon. They are as follows: GIPZ empty vector, Human Cofilin A V2LHS_64316, Human Cofilin B V2LHS_64314, Mouse Cofilin A V3LMM_449309, Mouse Cofilin B V3LMM_521443. Using Xtreme Gene HP DNA Transfection Reagent (Version 08, Roche), these vectors were transfected into 293T cells with pMD2.G (Addgene #12259) and psPAX2 (Addgene #12260) to package lentivirus. Lentiviral stocks were filtered by 0.45 μm polyethersulfone membranes (VWR 28145–505). Cancer cells were incubated with eGFP::cofilin lentivirus and 4 μg ml^−1^ polybrene for 8 h. After a 72 h culture period, successfully labelled cells were isolated by puromycin selection and knockdown confirmation with qPCR and western blot. siRNA targeting cx43 was transfected using Lipofectamine RNAiMAX Transfection Reagent (Thermo Fisher #13778). Cells were allowed to stabilize after transfection for 48 h, then seeded for migration assays. For western blots, the Electrophoresis and Membrane-Transfer were performed by iBlot (Invitrogen). Imaging was obtained by Odyssey (Li-Cor) system, following the manufacturer’s protocol.

### Cell culture procedures

MCF7, MDA-MD231, SCP28, 4T1, 4T1.2, and Raw 264.7 were cultured in DMEM with 10% (vol/vol) bovine calf serum and 1% pen strep amp at 37 °C and 5% CO2. T47D ZR-75-1 were cultured in RPMI-1640 at 37 °C and 5% CO2 (T47D media was supplemented with 0.2 Units/ml bovine insulin). hFOB 1.19, MSCs, MC3T3-E1 were cultured in phenol red-free DMEM/F12 a:1 with 10% (vol/vol) bovine calf serum and 1% pen strep amp at 34 °C and 5% CO2. T47D ZR-75-1 at 37 °C and 5% CO2.

### Migration assays

All co-culture experiments were in phenol red-free DMEM/F12 1:1 with 0.2% FBS and 1% pen strep amp at 37 °C and 5% CO2. Removable silicone culture-inserts were purchased from Ibidi-Fisher Cat. No: 80369. For 2D cell migration assays, 5000 cells per cell type were seeded into silicone chamber and allowed to attach for one hour before removal, and imaged over 48 h in a Sartorius IncuCyte S3 long term live imager at ×4 magnification. Cells were counted in fields sized 1.25 mm by 1.00 mm immediately adjacent to removed silicone barrier (long edge parallel to removed barrier), chosen at random but fully flush to edge. Three fields per well were averaged for a single replicate. The researcher was not blinded to treatment group during quantification. For transwell assays, 10,000 cells per cell type were seeded into the top chamber in 100 µL of co-culture medium, and allowed to attach for one hour before the addition of 650 µL chemoattractant/drug-containing co-culture media to the bottom well. 6.5 mm inserts with a pore size of 8 µm were purchased from Corning (3422). Human Bone Morphogenetic Protein-2 (BMP-2) was purchased from Alfa Aesar™/Fisher (J67332EXE). Epidermal growth factor (EGF) Recombinant Human Protein was purchased from Thermo Fisher Scientific (PHG0311L). At 48 hours, cells were scraped from the top of the insert with a cotton swab, then the inserts were fixed in 5% glutaraldehyde for 10 minutes, and finally rinsed with water. Transwell cells were counted using fluorescence on a Leica DMI8 with a 20×/NA objective. Three fields per insert were chosen at random and averaged to form a single replicate. The researcher was not blinded to treatment group during quantification. For anti-proliferation control (Supplementary Fig. 1f) 2 uM thymidine was added. For extracellular matrix control (Supplementary Fig. 1h), osteoblasts were incubated with the transwell inserts for 24 h before trypsin-free removal with EDTA, and the addition of cancer cells. Live imaging chemotaxis was captured using Incucyte ClearView plates and imaged every 2 h for a total of 70 h. Raw data available online^47^.

### Live-cell microscopy

Live cell imaging was performed with several different technologies. Time lapse imaging was achieved using an Incucyte S3 at 4x or 10x magnification at 37 °C and 5% CO2. Images were taken every hour for a minimum of 48 h and combined to form time-lapse videos. Biotek Cytation 5 Epi-fluorescence microscope time lapse imaging and GE Healthcare Deltavision DV Live imaging were done with the assistance of the Integrated Microscopy Core at Baylor College of Medicine. Both of these imagers utilized environmental control at 37 °C and 5% CO2. Image frequency varied by experiment. eGFP::cofilin live imaging was performed on a Nikon Eclipse TS100 with DS-Fi2 camera.

### Immunofluorescence microscopy

Cells seeded onto Nunc™ Lab-Tek™ II CC2™ 4-well chambers slides were fixed with 4% paraformaldehyde for 10 min at 0 °C and permeabilized using 100% ethanol at 0 °C for 10 min. Samples were then blocked with 5% normal goat or donkey serum in 10% BSA PBST (PBS + 0.2% (vol/vol) Triton X-100). Primary antibody labeling was performed in 10% BSA PBST overnight at 4 °C (1:1000), while secondary antibody (1:250) incubation was for 2 h at room temperature in the same solution. After DAPI stain there samples were mounted with ProLong gold antifade (Thermo Fisher Scientific, P36930). Imaging was performed on a GE Healthcare DeltaVision LIVE Deconvolution Microscope with the assistance of the Integrated Microscopy Core at Baylor College of Medicine. Imaging was performed using an Olympus UApo 40×/1.35 objective, z stacks were acquired at 0.4 µm optical spacing covering the whole of the cells in field before applying a conservative restorative algorithm for quantitative image deconvolution in SoftWorx v7.0, 10 cycles were used for all the images. Max intensity projections were then generated and used in the figures.

For mouse bones immunolabeling, the harvested hind limb bones were fixed in 4% PFA overnight then decalcified in 14% EDTA over the course of a week. Bones were then embedded in paraffin and sectioned with assistance by the Baylor College of Medicine Breast Center pathology core. Sections were treated in a 55 °C oven for 1–2 h, then incubated in xylene 3 times for five minutes each, before rehydration in 3 min incubations in 100%, 95%, 70%, 50% ethanol and finally water. Antigen retrieval was performed by 10 m, 95 °C citric acid treatment. After PBS washes, slides were blocked in 10%serum in PBST. Primary treatment was incubation in primary antibody diluted with 1.5% serum (donkey and/or goat) in PBST overnight at 4 °C. Secondary antibody treatment was 30 min (1:250 alexa 488, 594) diluted in PBST. Slides were treated in 1:1000 DAPI for 10 min and sealed under cover glass with anti-fade solution and nail polish.

### Correlative light and electron microscopy and cryoET

QUANTIFOIL® Gold 200 mesh london finder grids with an R2/2 holey carbon film of round 2 µm holes with 2 µm spacing (Quantifoil Micro Tools GmbH) were placed in the 10 mm glass well of a MatTek 35 mm cell culture dish, then coated with collagen overnight. Red-labeled MCF-7 cells and GFP-labeled osteoblast pairs sorted by FACS were plated and grown on the grids overnight. Grids were then transferred to the blotting chamber of a Leica EMGP vitrification device at 37 °C and 90% humidity. Inside the blotting chamber, 3 µl of BSA-treated 15 nm gold fiducial marker solution (Electron Microscopy Sciences Cat# 25489) was added to the sample-side of the grid immediately prior to blotting from the back-side of the grid using Whatman 541 ashless filter paper (GE Healthcare part #1541055) and plunging into nitrogen-cooled liquid ethane at −168 °C. Blot times of grids with successfully vitrified cells ranged from 3–5 s.

Tilt series were collected at 200 kV on a JEOL JEM2200FS energy-filtered field-emission cryo-electron microscope using a Gatan Model 895 UltraScan 4000 4k × 4k CCD camera and a Gatan Model 626 side-entry cryo-specimen holder (Gatan, Inc.). The nominal magnification used for imaging was ×15,000 with a calibrated pixel size of 7.64 Å/pixel. Data collection was performed using SerialEM^48^ with a target defocus of −6 µm, a tilt range of −60° to +60° 3 degree increments, and a total electron dose of ~60 electrons/Å2. Fiducial-marker-based tilt-series alignment and 3-dimensional tomographic reconstruction by back-projection were performed using IMOD^49^. Annotation of cellular features was performed manually using Amira (Thermo Fisher Scientific).

### Flow cytometry

Flow cytometry was performed as previously described. Calcein Red loaded MCF-7 cells and GFP-labeled osteoblasts were co-cultured for 18 h before being captured by trypsin, washed, and analyzed with BD FACS Aria II with help from the Baylor College of Medicine Cytometry and Cell Sorting Core.

### Animals

C57B/6, NRG, athymic nude mice, Sp7-tTA,tetO-EGFP/cre (OSX-GFP) mice, B6.Cg-Tg(Cspg4-cre/Esr1*)BAkik/J (NG2-CreER), and B6.Cg-Gt(ROSA)^26Sortm14(CAG-tdTomato)Hze^/J (Ai14D) mice were purchased from Jackson Laboratory. NG2-RFP mice were generated by crossing NG2-CreER and Ai14D mice. The strain and number of mice used for each experiment are mentioned in text and legends. All animal work was done in accordance with a protocol approved by the Baylor College of Medicine Institutional Animal Care and Use Committee. The investigator was not blinded to the group allocation during the whole experiment.

### Ex vivo bone imaging

Intra-iliac injections and mammary gland injections were performed as previously described. Mice were sacrificed 24 h after IIA injection and the femur and tibia were harvested, cut into 1–2 mm fragments and cultured in DMEM 10% FBS media, imaged daily. For spontaneous metastasis, mammary gland injected tumors were allowed to grow to 1 cm diameter, then resected. Mice were sacrificed after about 18 days before bones were harvested, cut into fragments, and cultured. Bone fragments were imaged on both Echo Revolve R4 and GE Healthcare Deltavision DV.

### Statistics

We used analysis of variations (ANOVA) and analysis of covariance (ANCOVA) to compute statistical significance based on data generated by independent experiments. In both cases, normalized numbers of cell migration (cell counts over area of the field) were used as response. In BMP-2-chemotaxis transwell migration assays, the covariates include BMP-2 concentrations as a continuous variable. Other variables, including the presence of OBs, knockdown of cofilin, and treatment of CBX are categorical. Importantly, independent experiments were also included as categorical variables to control for inter-experimental variations. A generalized linear model was then fit using the “lm” package of R. The reported *p*-values were computed between the experimental groups indicated by the straight lines in each figure with covariate of independent experiments. For DSLS+ proportion, error calculated by binomial distribution.

### Reporting summary

Further information on research design is available in the Nature Research Reporting Summary linked to this article.

## Supplementary information

Muscarella et al Supplementary Information

Dataset 1

Dataset 2

Dataset 3

Supplementary Video 1

Supplementary Video 2

Supplementary Video 3

Supplementary Video 4

Supplementary Video 5

Supplementary Video 6

Reporting Summary Checklist FLAT

## Data Availability

The data generated and analysed during this study are described in the following data record: 10.6084/m9.figshare.12682523^47^. This data record contains all data and supplementary data referred to from this paper.
