# Supplementary material for: Unique cellular protrusions mediate breast cancer cell migration by tethering to osteogenic cells
Source: NPJ Breast Cancer. 2020 Sep 7;6:42. doi: 10.1038/s41523-020-00183-8 (PMC7477119; doi:10.1038/s41523-020-00183-8)
Supplement: Supplementary file 1 — Muscarella et al Supplementary Information [file 41523_2020_183_MOESM1_ESM.pdf]

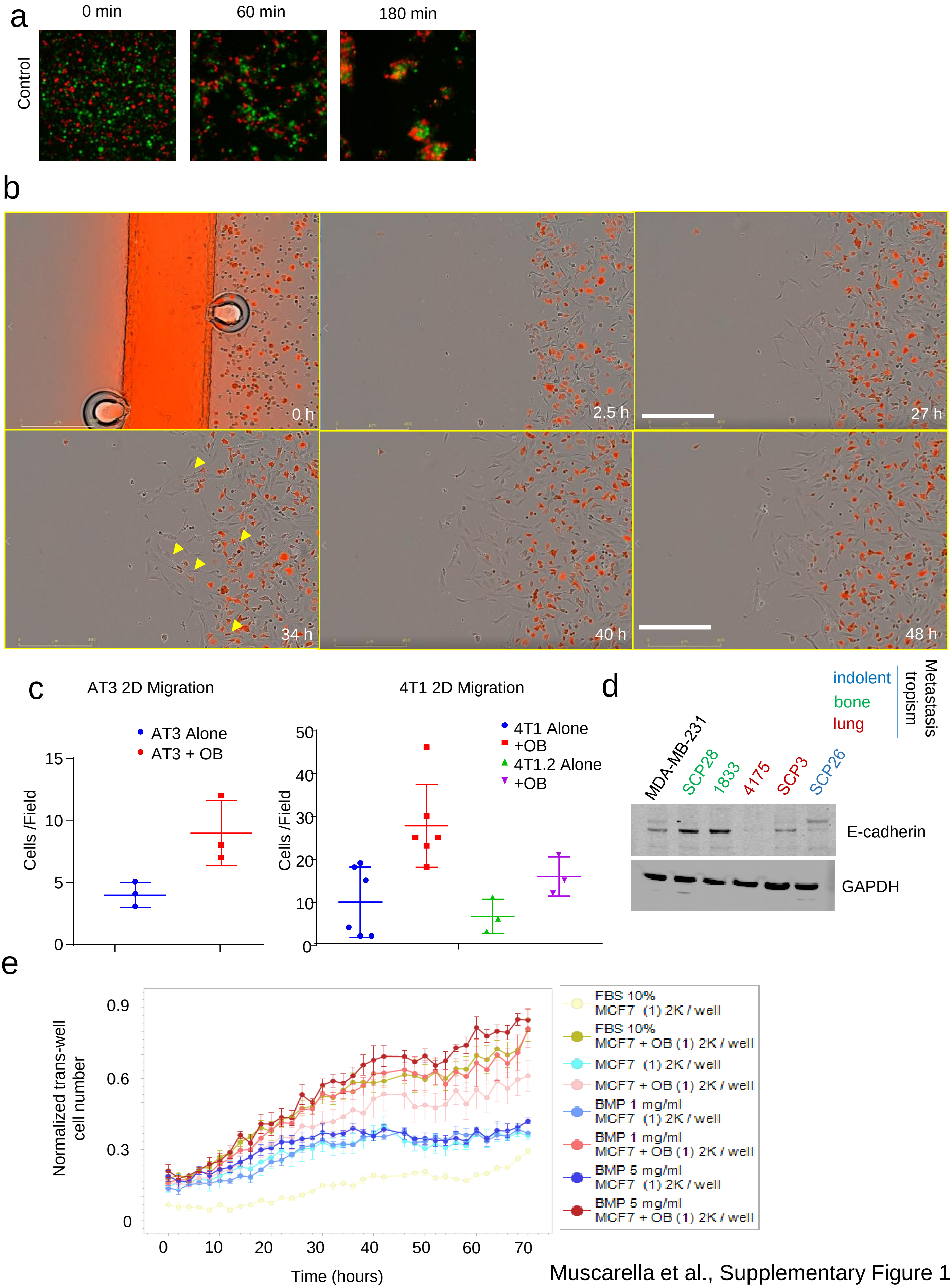

Muscarella et al., Supplementary Figure 1

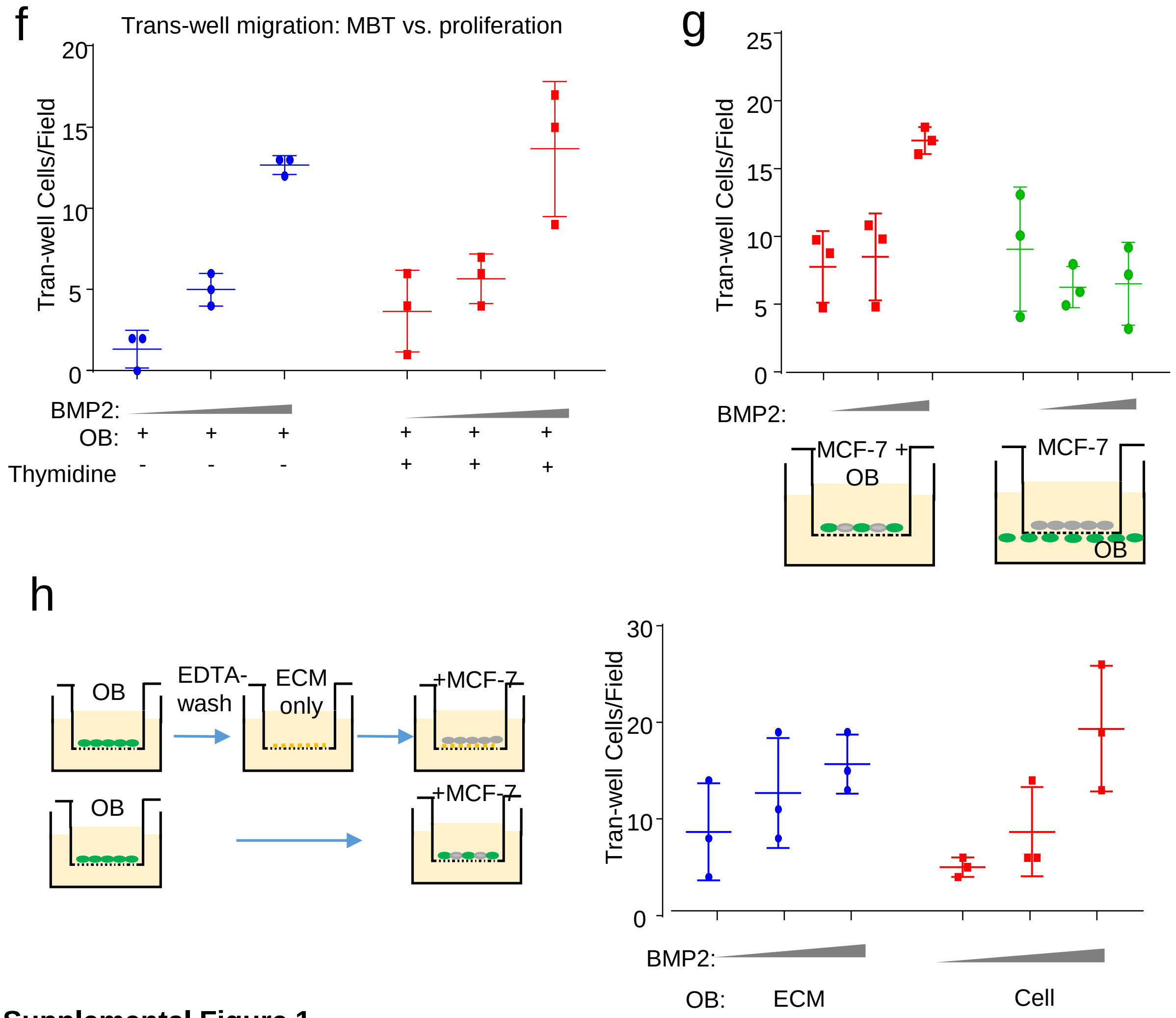

Supplemental Figure 1

a) Breast cancer cells (represented by MCF-7, RFP-tagged) readily form heterotypic organoids with osteoblasts (OBs, GFP-tagged) in 3D suspension.

b) Time lapse of MCF7/OB coculture within silicone chamber and 48hr coculture. MCF7 cells bind and co-migrate into free space with osteoblasts (yellow arrows). Scale bar is 400µm.

c) Dot plot shows the number of cells migrated across borders between two chambers in the 2D migration assay shown in Fig. 1A. Mouse mammary tumor lines AT3, 4T1, and 4T1.2 were examined either alone and with OBs after 48 hours.

d) Western blot shows MDA-MB-231 expresses low level E-cadherin. Bone-tropic sublines SCP28 and 1833 express an increased level of E-cadherin in comparison to the parental line and other MDA-MB-231 sublines with different metastasis organotropism. Blot derives from the same experiment and that they were processed in parallel.

e) Incucyte S3 live trans-well quantification in ClearView chamber. The addition of OBs increases migration through the trans-well pore in a BMP2 dose-dependent fashion. 10% FBS used as a positive control. Values are normalized to initial top-well cell count. Each dot an average of three technical replicates.

f) Dot plot showing trans-well MCF-7 migration with and without 2uM thymidine treatment to halt proliferation.

g) Dot plot showing trans-well MCF-7 migration toward a gradient of BMP2, with OBs in the top or bottom chamber to distinguish MBT from paracrine-mediated chemotaxis.

h) Dot plot of MCF-7 trans-well migration in which OBs were cultured in the well for 24 hours, then either removed or allowed to remain, with MCF-7 seeded into both conditions to test if OB-produced extracellular matrix could be responsible for increased migration.

For all dot plots, each point represents a technical replicate, error bar indicates standard deviation.

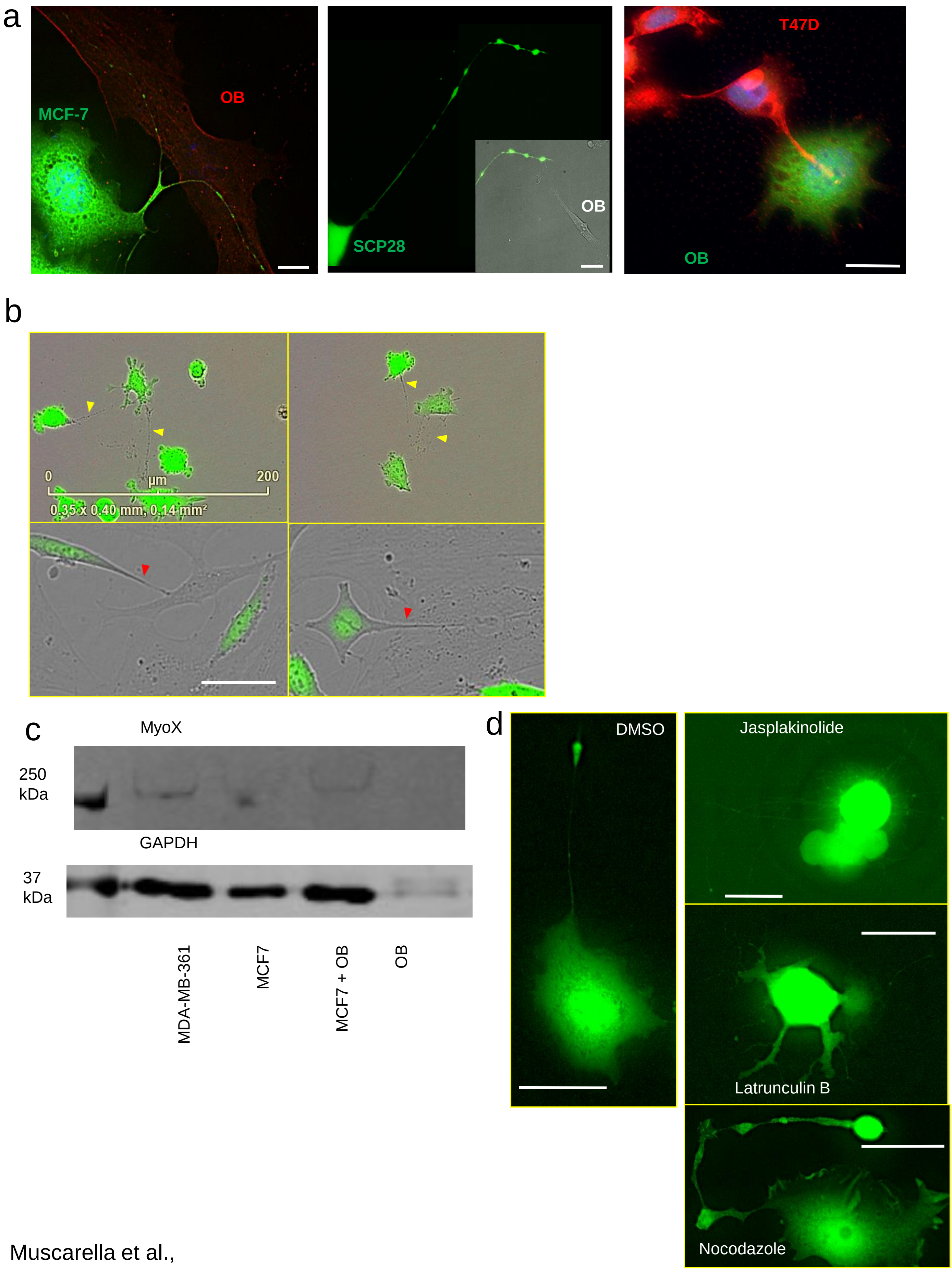

Muscarella et al.,
Supplementary Figure 2

Supplemental Figure 2

a) Multiple cell lines, MCF-7, SCP28, T47D respectively, with DSLS contacting OBs in co-culture. Scale bars are 5, 25, and 5µm respectively.

b) AT3 cells (Green) can express tunneling nanotubes (TNTs) on their own (top, yellow arrows). These are morphologically distinct from DSLS expressed when in coculture with mesenchymal stem cells (bottom, red arrow). Scale bars are 200 and 50µm respectively.

c) Western blot for Myosin X in MDA-MB-361, MCF7, MCF7+hFOB1.19 coculture, and hFOB 1.19 alone. MDA-MB-361 control was from a distinct experiment, MCF7, MCF7+hFOB1.19, and hFOB1.19 derive from the same experiment. Blots were processed in parallel.

d) MCF7 DSLS after treatment with 2mM Latrunculin B, 100 nm Jasplkinolide, and 250nM Nocodazole. Scale bars are all 20µm.

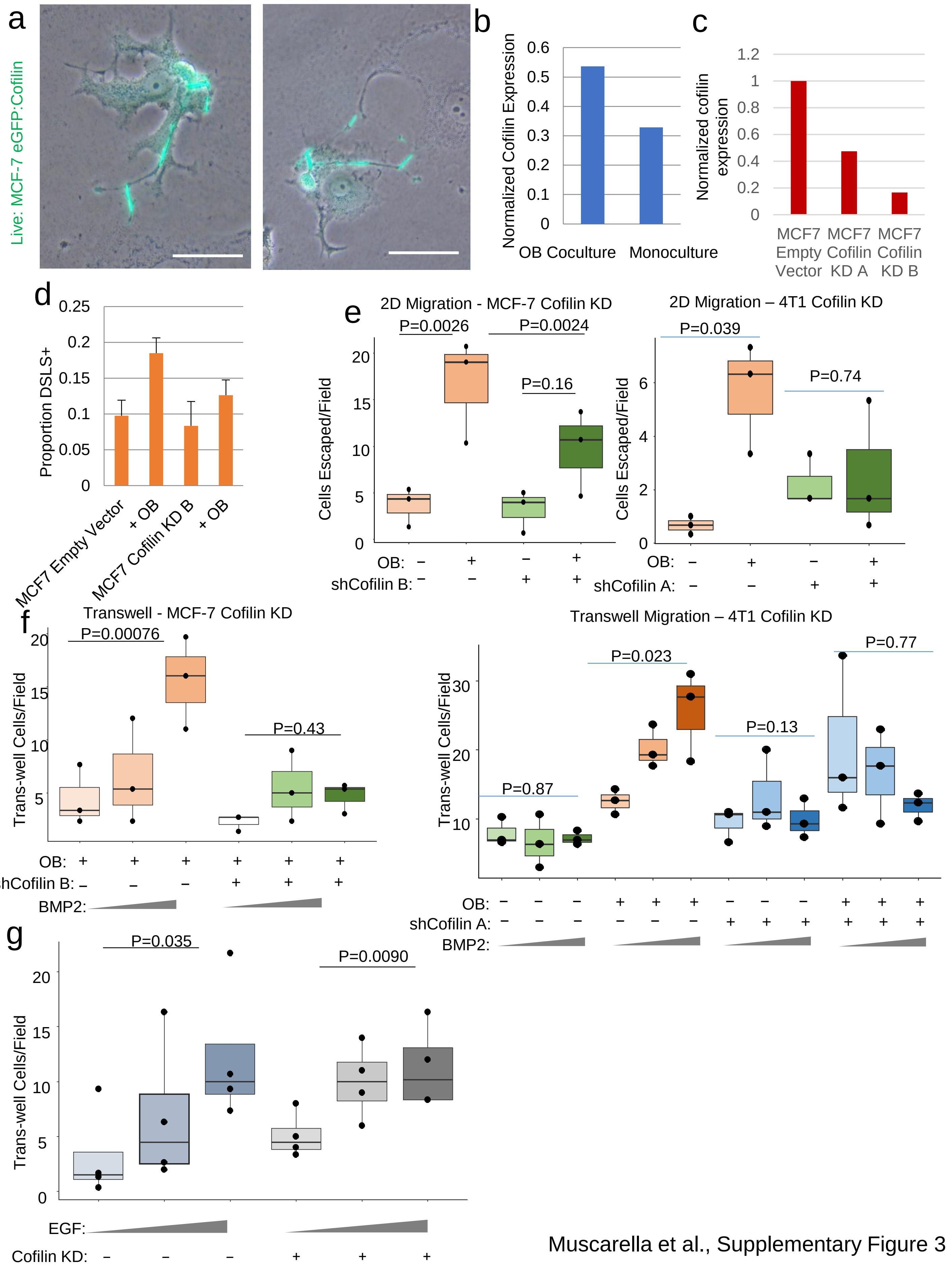

Muscarella et al., Supplementary Figure 3

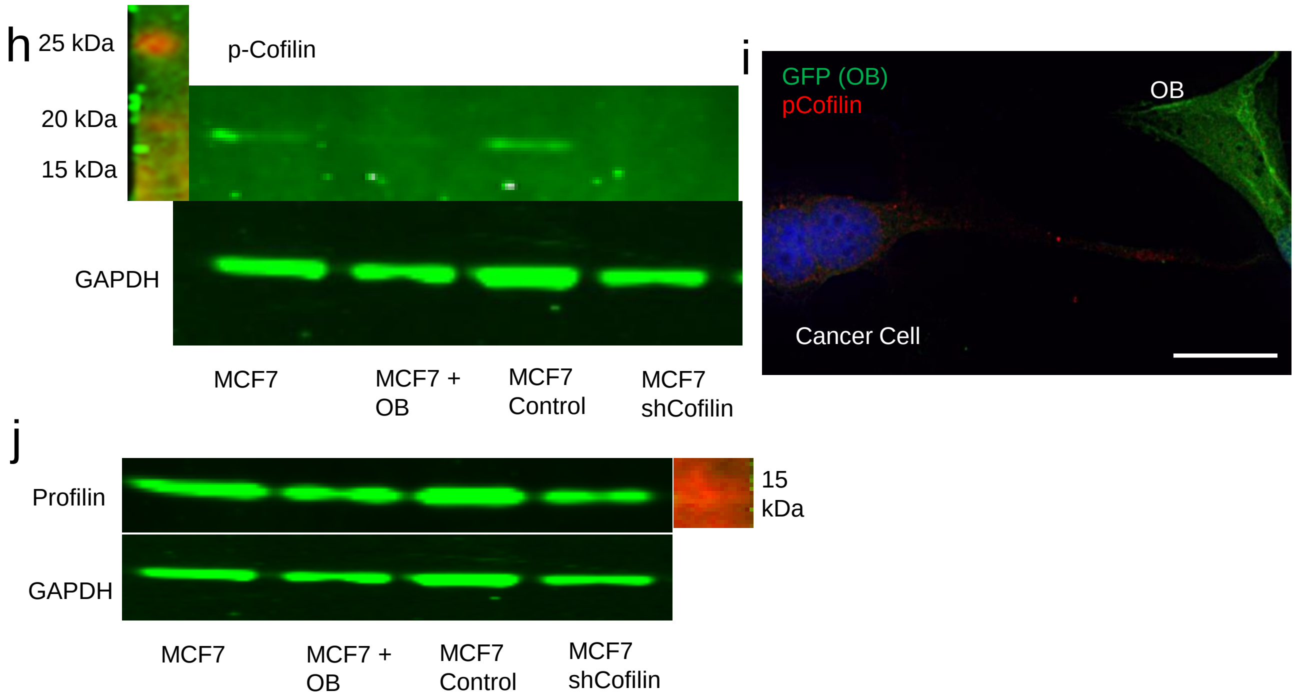

Supplemental Figure 3

a) MCF-7 transfected with constitutive eGFP:cofilin1 construct displays cofilin bands along the length of DSLS. Scale bars are 25 and 30µm respectively.
b) Quantification of cofilin expression by western blot for MCF-7 in monoculture vs co-culture with OBs, normalized to GAPDH expression.
c) Cofilin knockdown lines exhibit a minimum 50% reduction in baseline cofilin level (as determined by western blot, normalized to GAPDH), and in experimental conditions,
d) The proportion of cells with DSLS in MCF-7 cells with or without co-culture of OBs and with or without knockdown of cofilin. Error bars are based on 2 biological replicates.
e) Boxplots showing the 2D migration of MCF-7 and 4T1 cells across the chamber border with or without shRNA-mediated KD of cofilin (shCofilin).
f) Boxplots showing the trans-well migration of MCF-7 and 4T1 cells in response to BMP2 with or without knockdown of cofilin, and with and without OBs.
g) Boxplots showing trans-well MCF-7 migration toward and an EGF gradient under indicated setting of cofilin KD and CBX treatment.
h) Western blot showing phosphorylated cofilin (p-Cofilin) expression by MCF7 alone, with osteoblasts (MCF7 + OB), MCF7 shRNA control (empty vector) and with cofilin knockdown (shCofilin). “MCF7, MCF7 + OB” lanes are from same experiment, “MCF7 control”, “MCF7 shCofilin” lanes from a separate experiment. Blots processed in parallel.
i) Phosphorylated cofilin in MCF7 DSLS (red) contacting osteoblast (green). Scale bars is 30µm.
j) Western blot showing profiling expression by MCF7 alone, with osteoblasts (MCF7 + OB) MCF7 shRNA control (empty vector) and with cofilin knockdown (shCofilin). “MCF7, MCF7 + OB” lanes are from same experiment, “MCF7 control”, “MCF7 shCofilin” lanes from a separate experiment. Blots processed in parallel.

For d), error bars indicate standard deviation.

For e)-g), each point represents an biological replicate, an average of three technical replicates. For box plots, the lower and upper hinges correspond to the first and third quartiles (the 25th and 75th percentiles). The upper whisker extends from the hinge to the largest value no further than 1.5 * IQR from the hinge. The lower whisker extends from the hinge to the smallest value at most 1.5 * IQR of the hinge. Data beyond the end of the whiskers are called "outlying" points and are plotted individually. P-value as determined by ANOVA (e) or ANCOVA (f, g) as elaborated in Methods.

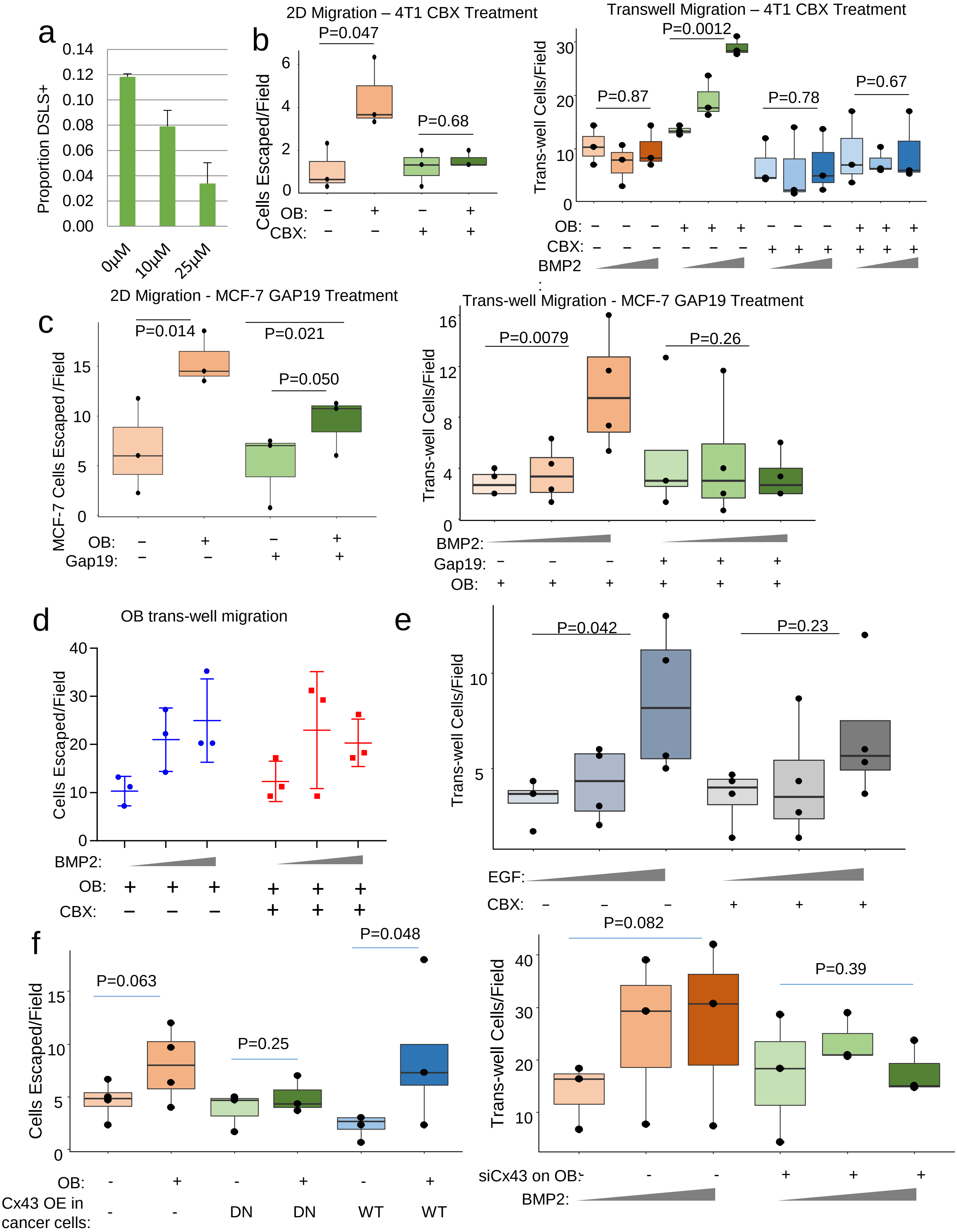

Muscarella et al., Supplementary Figure 4.

g

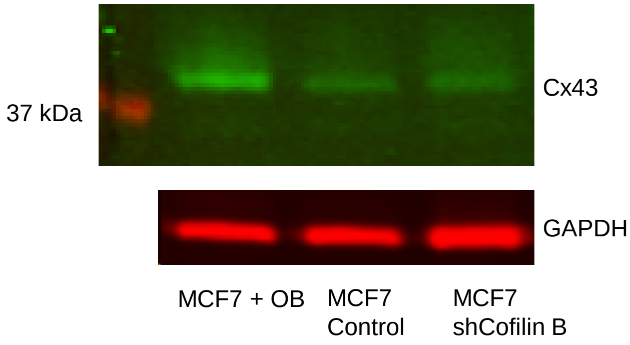

Supplementary Figure 4

a) Proportion of MCF7 cancer cells with DSLS under indicated concentration of CBX while in coculture with osteoblasts.
b) Box plots showing 2D and 3D migration for 4T1 cells with and without osteoblasts and 10µM CBX treatment.
c) Box plots showing the 2D migration cells across the chamber border (left) or cells fulfilled trans-well migration in response to BMP2 (right) for MCF-7 treated with 25µM GAP19, a Cx43-specific blocking peptide, with and without OBs.
d) Dot plot showing the trans-well migration of OBs in response to variable concentration of BMP2 with or without CBX treatment.
e) Box plots showing the trans-well migration of MCF-7 cells in response to variable concentration of EGF with or without CBX treatment.
f) Box plots showing the 2D migration and transwell migration of MCF7 cells with untreated osteoblasts and osteoblasts transfected with Cx43-targeting siRNA.
g) Western blot of Cx43 in MCF7 cells with cofilin knockdown. OB: osteoblasts. KD B: #B shRNA against Cx43. Blot derives from the same experiment and that they were processed in parallel.

For a) and d) error bars indicate standard deviation
For b), c), e), and f), each point represents an biological replicate, an average of three technical replicates. For box plots, the lower and upper hinges correspond to the first and third quartiles (the 25th and 75th percentiles). The upper whisker extends from the hinge to the largest value no further than 1.5 * IQR from the hinge. The lower whisker extends from the hinge to the smallest value at most 1.5 * IQR of the hinge. Data beyond the end of the whiskers are called "outlying" points and are plotted individually. P=Value as determined by ANOVA (b -2D, c-2D, f-2D) or ANCOVA (b – Transwell, c – Transwell, e, f - Transwell) as elaborated in Methods.

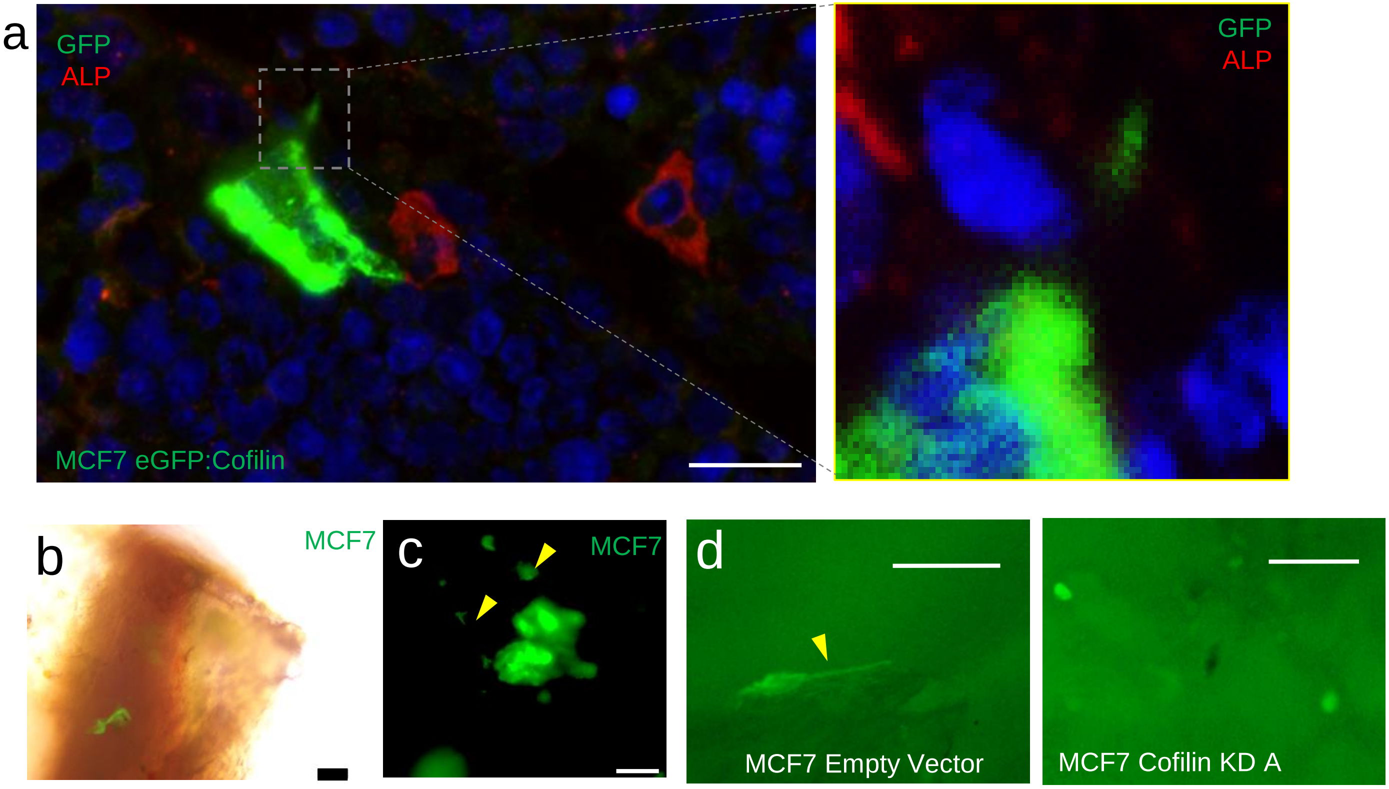

Supplemental Figure 5

a) eGFP:cofilin-tagged MCF7 cells were injected via Intra-Iliac Artery Injection (IIA Inj.) into nude mice. Mice were sacrificed at 72 hours. Immunofluorescence staining in femur shows cofilin bands present in the cellular protrusion. Scale bar is 15µm.

b) MCF7 cells were injected via Intra-Iliac Artery Injection (IIA Inj.) into nude mice. Mice were immediately sacrificed, hind limb bones harvested, and cut into small (1-2mm diameter) pieces. Bone fragments are cultured over the next 3 days and live-imaged each day. Day 1 bone fragment with eGFP:cofilin-tagged MCF7 cells detected through deconvolution microscopy. Scale bar is 50µm.

c) A high-resolution image of microscopic lesions of eGFP:cofilin-tagged MCF7 in bone. Yellow arrows indicate possible DSLS structures. Scale bars is 25µm.

d) GFP-tagged MCF7 Empty Vector GIPZ shRNA (left) and cofilin knockdown GIPZ shRNA (right) in the bone 24 hours after IIA injection and bone harvest. Yellow arrows indicate possible DSLS structures. Scale bars are 50µm.

1

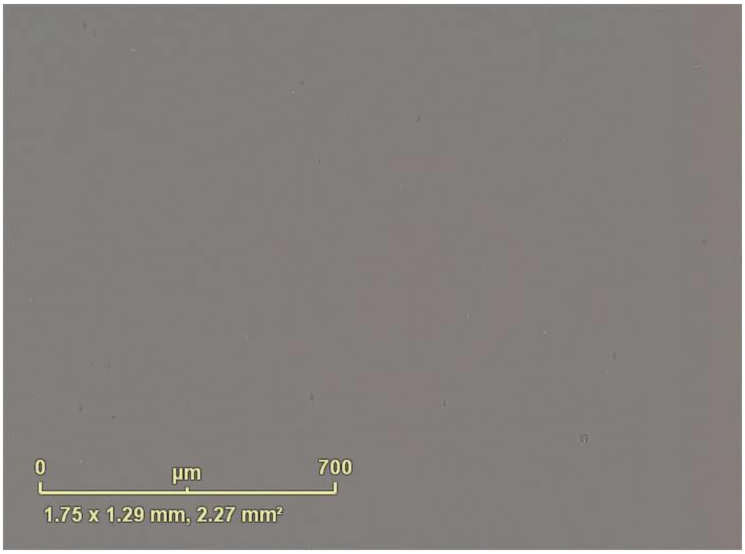

Supplemental Video 1

Time lapse of MCF-7 (red) originating in Ibidi silicone insert imaged every 45 minutes. MCF-7 migrate very little out of the original starting area.

2

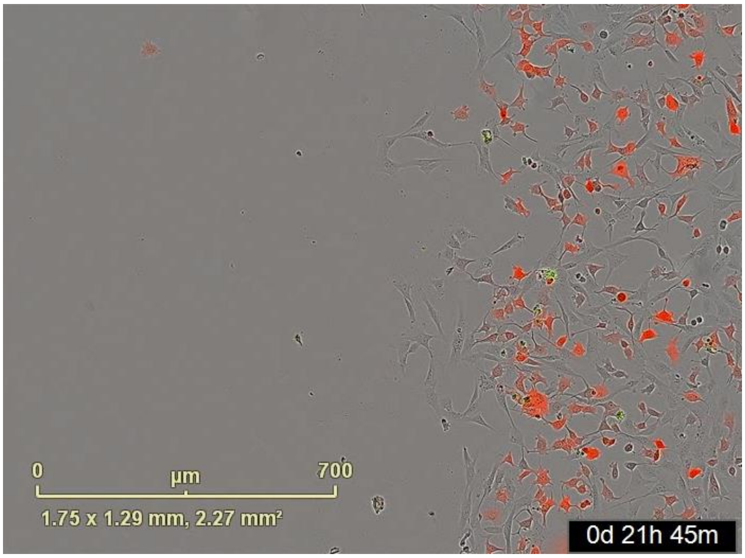

Supplemental Video 2

Time lapse of MCF-7 (red) and unlabeled OBs originating in Ibidi silicone insert imaged every 45 minutes. MCF-7 with DSLS can be seen migrating out of original area attached to OBs.

3

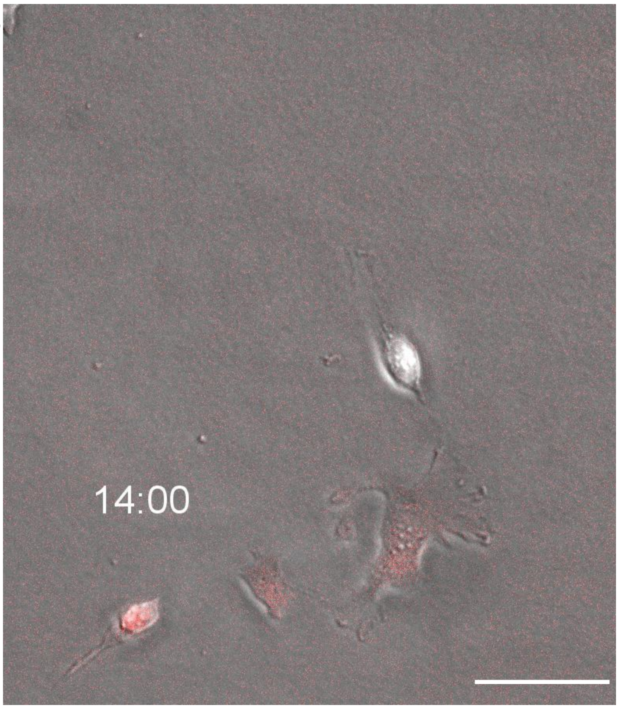

Supplemental Video 3

Video showing MCF-7 (red) making contact with hFOB 1.19 OBs and DSLS being pulled out of cancer cell. Scale bar is 150µm.

4

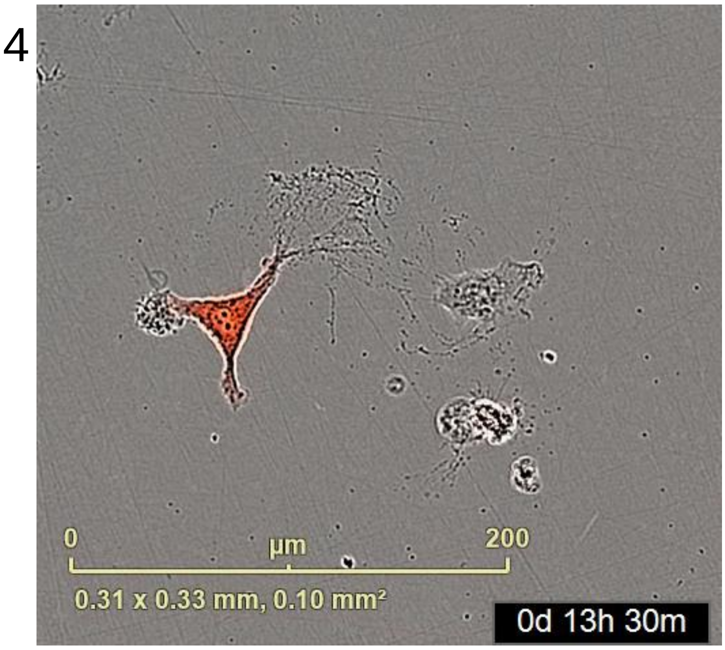

5

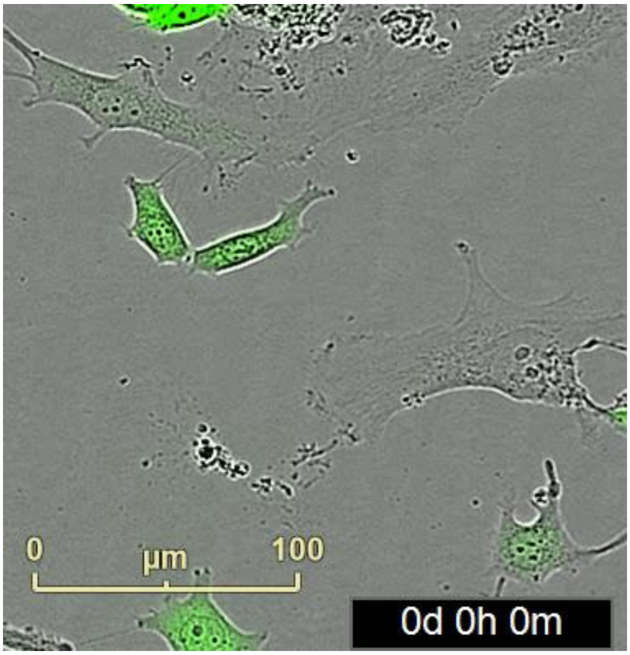

6

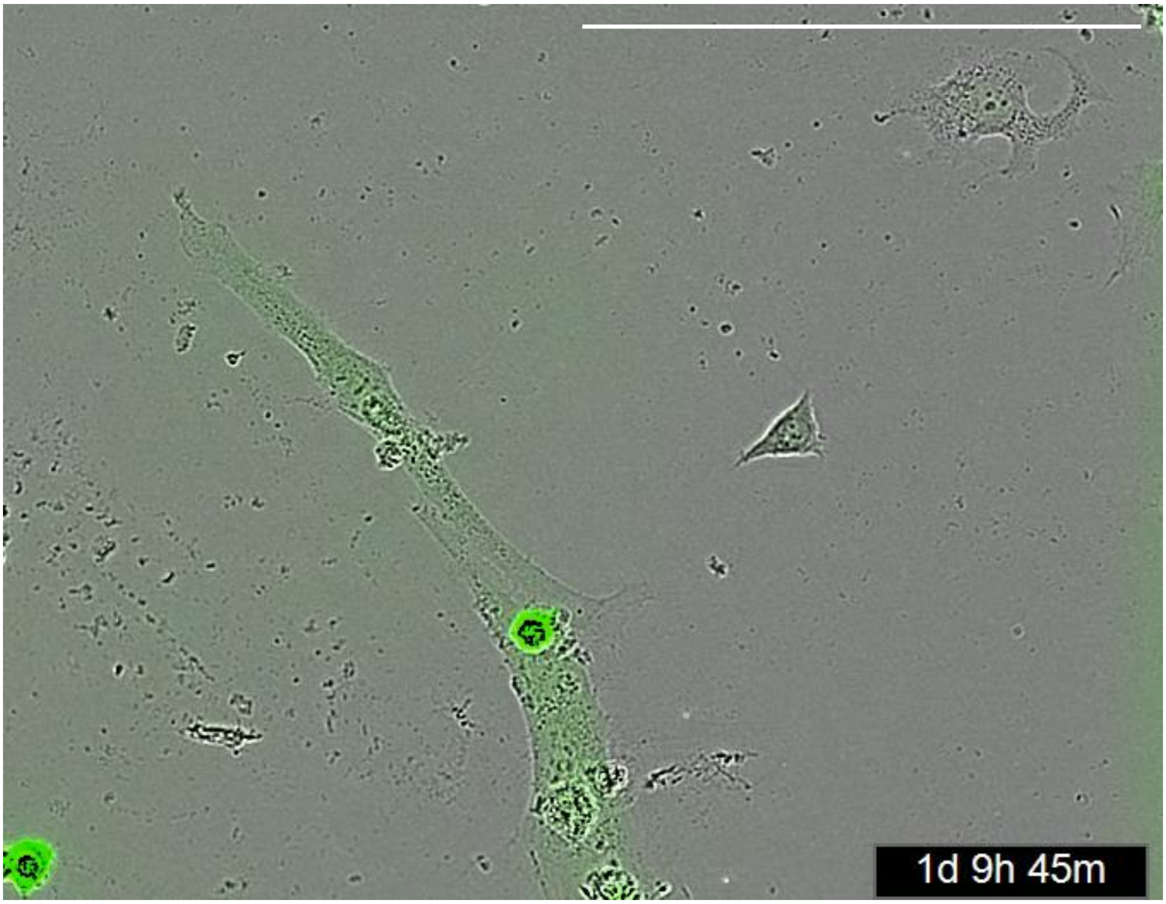

Supplemental Video 4

MCF-7 (red) latches onto migrating OBs, exhibits DSLS and migrates with OBs

Supplemental Video 5

MCF-7 (green) latches onto migrating OBs, exhibits DSLS and migrates with OB

Supplemental Video 6

MCF-7 labeled with eGFP:Cofilin fusion protein latches onto migrating OBs, forms protrusion with cofilin banding, and remains attached. MCF-7 migrates with OBs. Scale bar is 200µm.

Raw data for all numerical experiments can be found in the figshare repository. Data for Supplementary Figure 1e is found in “Incucyte Time-lapse Transwell Data.txt”, flow cytometry parameters can be found in “FlowCytometryParameters.wsp”. All other numerical data for the main figures and supplementary figures can be found in “Muscarella et al Raw Data.xlsx”.

Muscarella, A. M. et al. Metadata record for the manuscript: Unique gap junction- and cofilin-dependent cellular protrusions mediate cancer cell migration by tethering to osteogenic cells. figshare https://doi.org/10.6084/m9.figshare.12682523 (2020).

Whole Blot Images
Supplementary Figure 1d

E-Cadherin

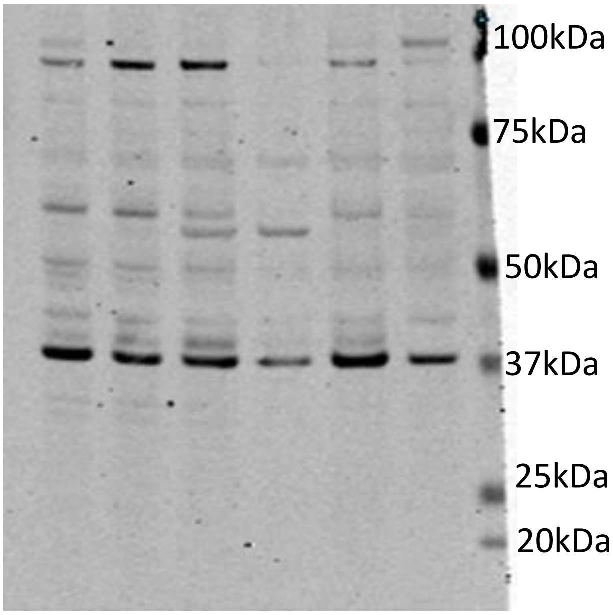

GAPDH

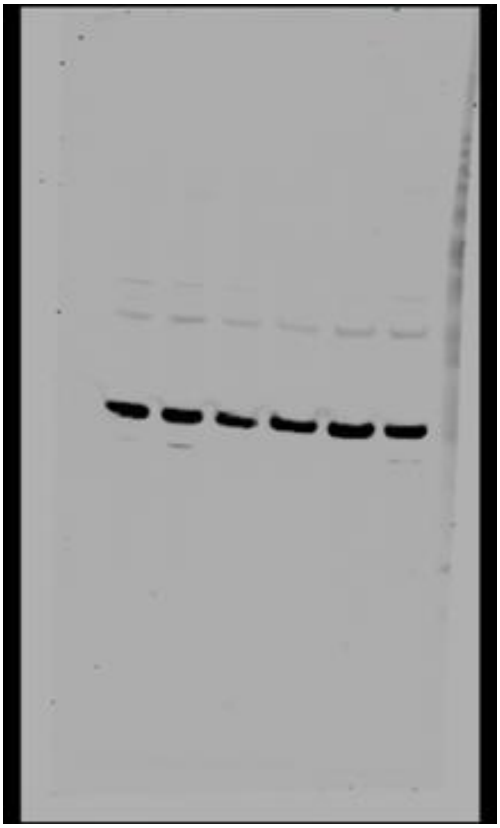

Supplementary Figure 2c

Myosin X

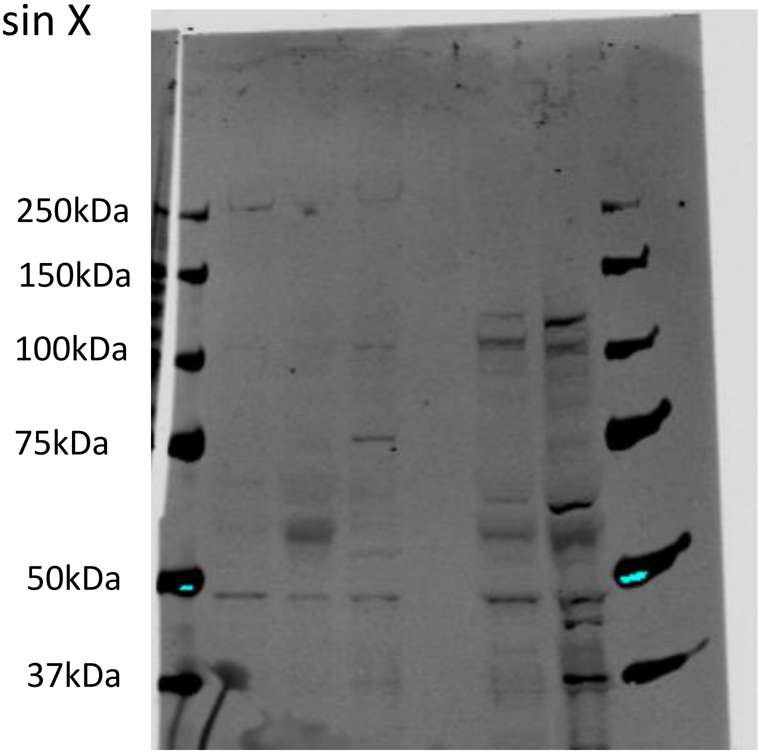

GAPDH

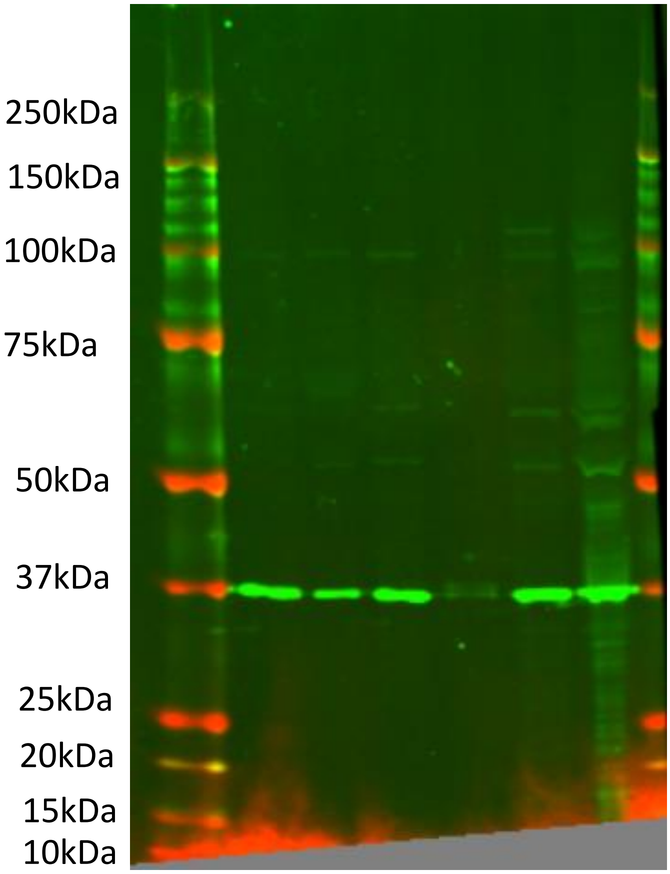

Supplementary Figure 3h Phospho-Cofilin

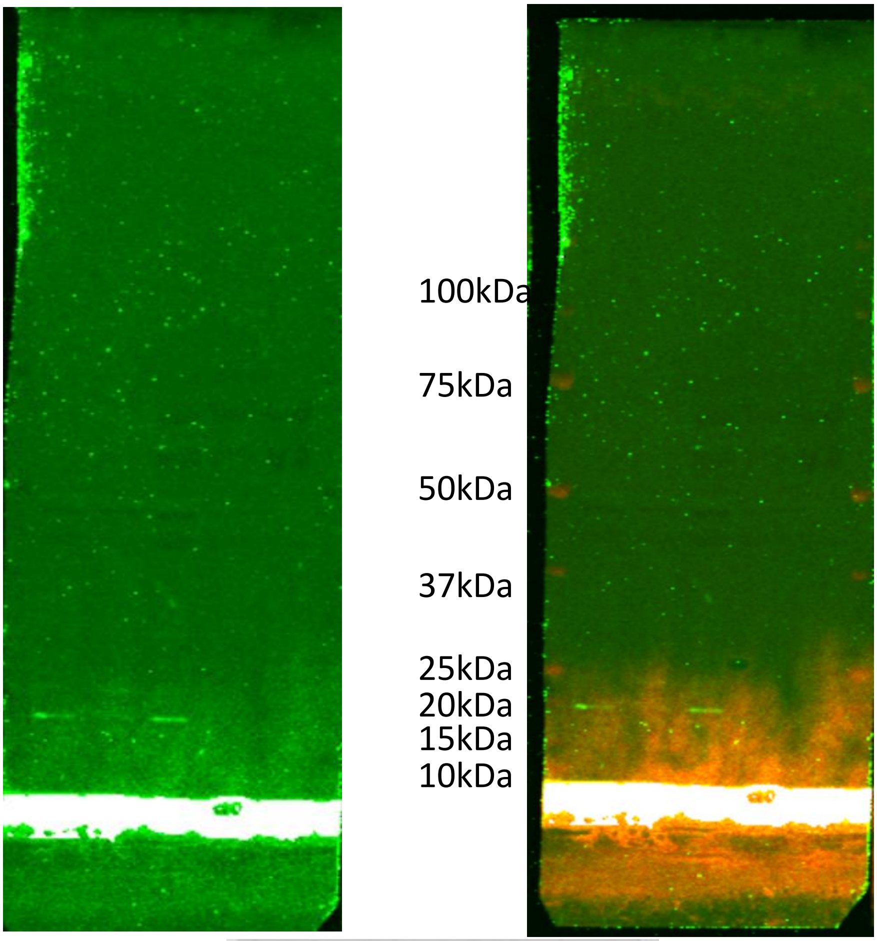

GAPDH

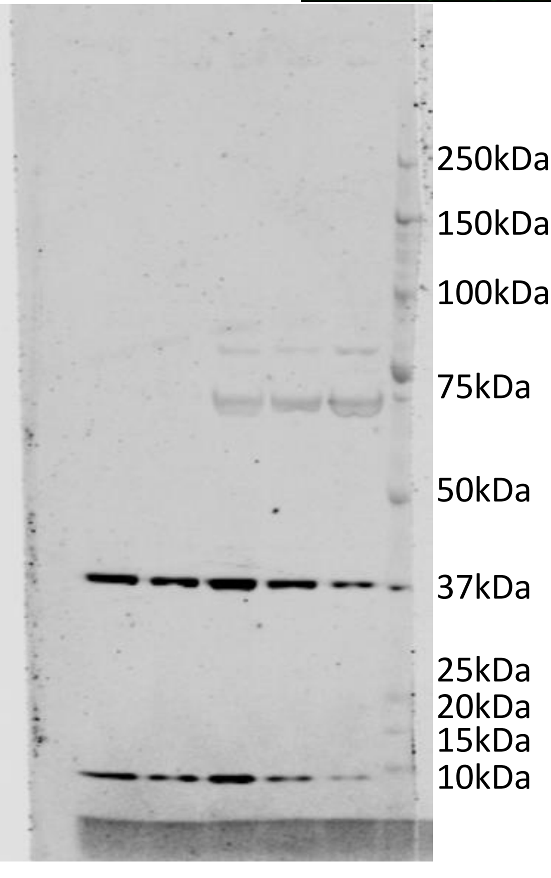

Supplementary Figure 3j

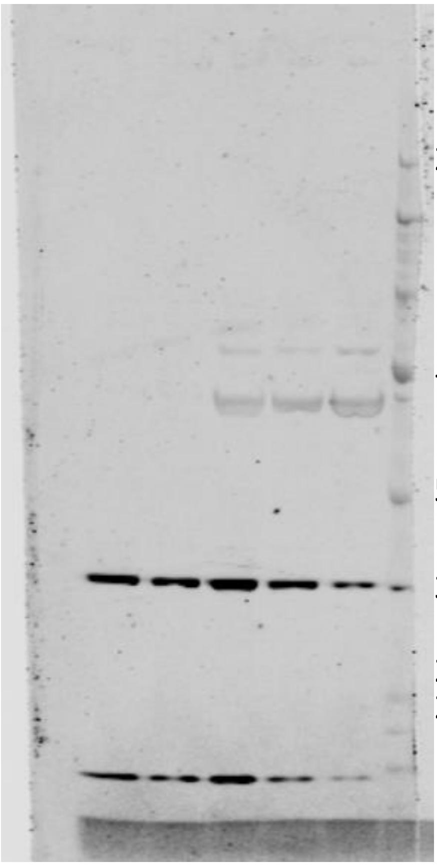

Supplementary Figure 4g

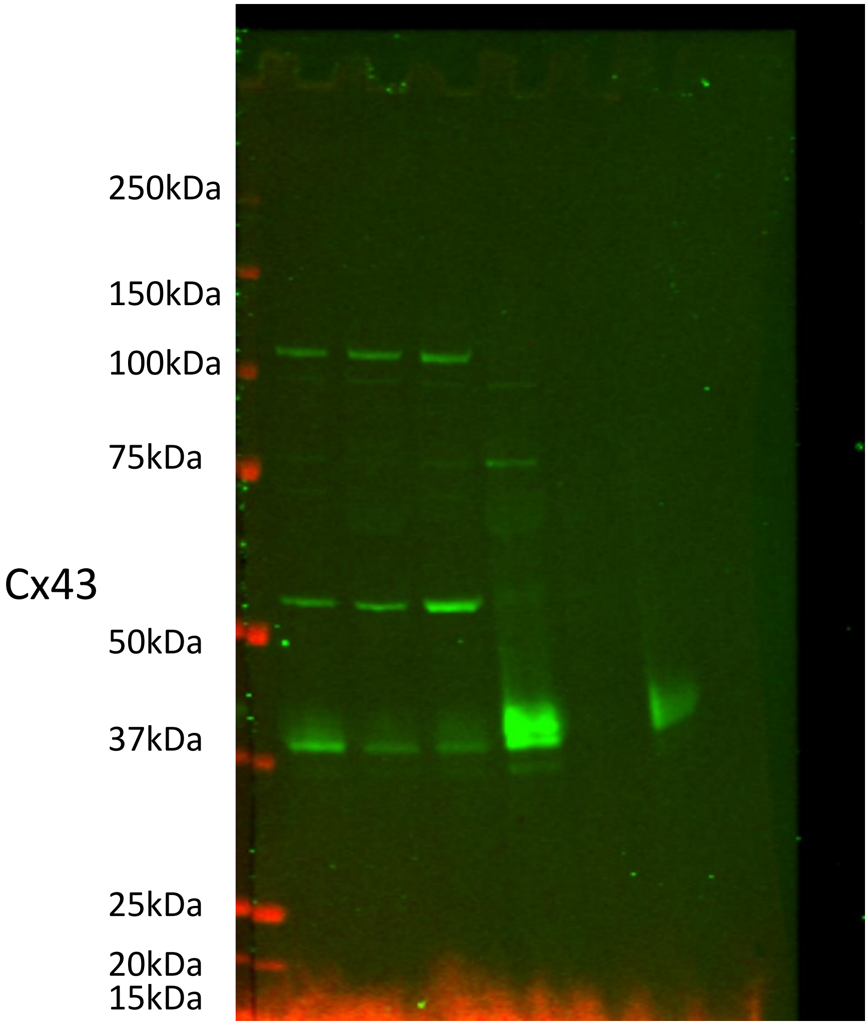

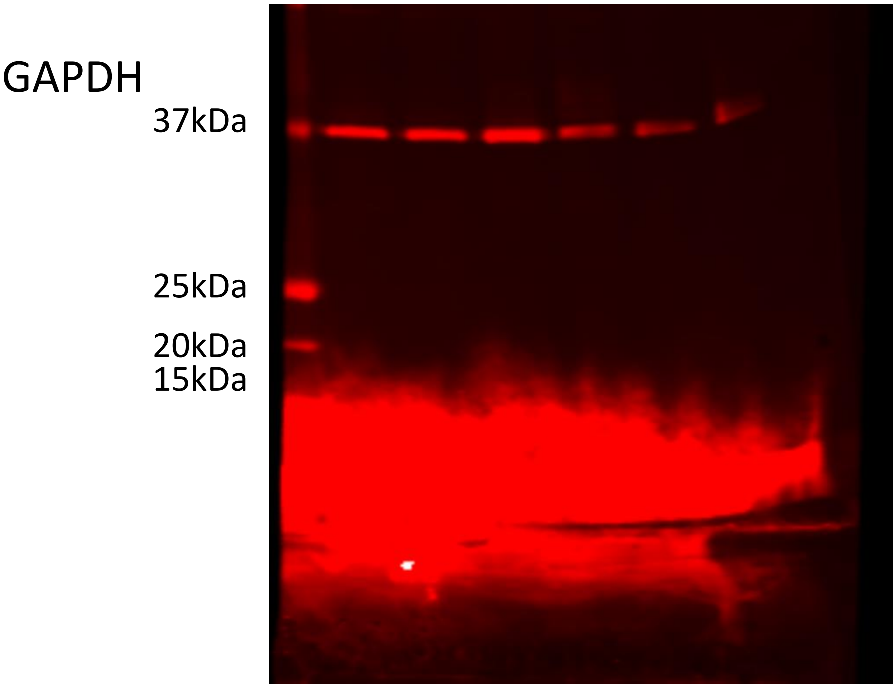

Cell Line	Subtype	DSLS
MCF10A	Normal Breast Epithelial Cell	-
MCF-12A	Normal Breast Epithelial Cell	-
T47D	Luminal	++
MCF-7	Luminal	+++
ZR-75-1	Luminal	+
HCC 202	Luminal/Her 2	-
MDA-MB-361	Her 2	-
HCC1954	Her2	+
HCC1937 (P21)	Basal/BRCA1	-
MDA-MB-231	Basal/Claudin Low	-
SCP28	Basal/Claudin Low	+
MDA-MB-436	Basal/Triple Negative	+
BT549	Basal/Triple Negative	-
AT3	Mouse Breast Epithelial	++
4T1	Mouse Breast Epithelial	++
4T1.2	Mouse Breast Epithelial	++
VCaP	Prostate Epithelial	++
DLD1	Colon Epithelial	-
Colo25	Colon Epithelial	-

Supplementary Table 1 – Cancer cell lines presenting with DSLS when cultured with osteoblasts.

Table of cancer lines examined for DSLS presence, in co-culture with OBs, rated by the approximate proportion of cells exhibiting the protrusions after an 18 hour incubation – (0%), + (0-5%), ++ (5-10%), and +++ (>10%). SCP28 and 4T1.2 represent bone tropic sublines of MDA-MB-231 and 4T1 respectively.
