# Supplementary material for: Unique cellular protrusions mediate breast cancer cell migration by tethering to osteogenic cells
Source: NPJ Breast Cancer. 2020 Sep 7;6:42. doi: 10.1038/s41523-020-00183-8 (PMC7477119; doi:10.1038/s41523-020-00183-8)
Supplement: Supplementary file 11 — Reporting Summary Checklist FLAT [file 41523_2020_183_MOESM11_ESM.pdf]

nature research

Corresponding author(s): Xiang H.-F. Zhang, PhD

Last updated by author(s): Aug 7, 2020

Reporting Summary

Nature Research wishes to improve the reproducibility of the work that we publish. This form provides structure for consistency and transparency in reporting. For further information on Nature Research policies, see our Editorial Policies and the Editorial Policy Checklist.

Statistics

For all statistical analyses, confirm that the following items are present in the figure legend, table legend, main text, or Methods section.

n/a	Confirmed	
☐	☒	The exact sample size (*n*) for each experimental group/condition, given as a discrete number and unit of measurement
☐	☒	A statement on whether measurements were taken from distinct samples or whether the same sample was measured repeatedly
☐	☒	The statistical test(s) used AND whether they are one- or two-sided<br>*Only common tests should be described solely by name; describe more complex techniques in the Methods section.*
☐	☒	A description of all covariates tested
☐	☒	A description of any assumptions or corrections, such as tests of normality and adjustment for multiple comparisons
☐	☒	A full description of the statistical parameters including central tendency (e.g. means) or other basic estimates (e.g. regression coefficient) AND variation (e.g. standard deviation) or associated estimates of uncertainty (e.g. confidence intervals)
☒	☐	For null hypothesis testing, the test statistic (e.g. *F*, *t*, *r*) with confidence intervals, effect sizes, degrees of freedom and *P* value noted<br>*Give P values as exact values whenever suitable.*
☒	☐	For Bayesian analysis, information on the choice of priors and Markov chain Monte Carlo settings
☒	☐	For hierarchical and complex designs, identification of the appropriate level for tests and full reporting of outcomes
☒	☐	Estimates of effect sizes (e.g. Cohen's *d*, Pearson's *r*), indicating how they were calculated

Our web collection on statistics for biologists contains articles on many of the points above.

Software and code

Policy information about availability of computer code

Data collection	ImageJ, ISartorius IncuCyte S3, Biotek Cytation 5, Deltavision SoftWorx v7.0, SerialEM, Amira
Data analysis	*Provide a description of all commercial, open source and custom code used to analyse the data in this study, specifying the version used OR state that no software was used.*

For manuscripts utilizing custom algorithms or software that are central to the research but not yet described in published literature, software must be made available to editors and reviewers. We strongly encourage code deposition in a community repository (e.g. GitHub). See the Nature Research guidelines for submitting code & software for further information.

Data

Policy information about availability of data

All manuscripts must include a data availability statement. This statement should provide the following information, where applicable:

- Accession codes, unique identifiers, or web links for publicly available datasets
- A list of figures that have associated raw data
- A description of any restrictions on data availability

All data generated or analyzed during this study are included in this published article (and its supplementary information files).

Field-specific reporting

Please select the one below that is the best fit for your research. If you are not sure, read the appropriate sections before making your selection.

☒ Life sciences ☐ Behavioural & social sciences ☐ Ecological, evolutionary & environmental sciences

For a reference copy of the document with all sections, see nature.com/documents/nr-reporting-summary-flat.pdf

Life sciences study design

All studies must disclose on these points even when the disclosure is negative.

Sample size	For migration assays, sample size was determined by microscope field view. For cell counting, a minimum threshold of 100 cells per technical replicate was used.
Data exclusions	N/A
Replication	Multiple independent biological replicates as indicated.
Randomization	Field views randomly selected.
Blinding	Investigator was not blinded, as the groups were with and without various cells that are immediately apparent to the viewer.

Reporting for specific materials, systems and methods

We require information from authors about some types of materials, experimental systems and methods used in many studies. Here, indicate whether each material, system or method listed is relevant to your study. If you are not sure if a list item applies to your research, read the appropriate section before selecting a response.

Materials & experimental systems

n/a	Involved in the study	
☐	☒	Antibodies
☐	☒	Eukaryotic cell lines
☒	☐	Palaeontology and archaeology
☐	☒	Animals and other organisms
☒	☐	Human research participants
☒	☐	Clinical data
☒	☐	Dual use research of concern

Methods

n/a	Involved in the study	
☒	☐	ChIP-seq
☐	☒	Flow cytometry
☒	☐	MRI-based neuroimaging

Antibodies

Antibodies used	Commercially available monoclonal antibodies (mAb) or polyclonal antibodies (pAbs) were used for Cofilin (rabbit pAb, Abcam ab42824), GFP (chicken pAb, Abcam ab13970), E-cadherin (rabbit mAb, Cell Signaling Technologies 3195S), N-Cadherin (mouse mAb, Abcam ab98952, Notch3 (rabbit pAb, Abcam ab23426), Cx43 (rabbit pAb, Sigma C6219), Myosin X (rabbit pAB, Novus, 22430002) and ALP (rabbit mAb, Abcam ab108337).
Validation	Positive control staining/western blot where applicable.

Eukaryotic cell lines

Policy information about cell lines

Cell line source(s)	All cell lines are originally derived from ATCC, except for 4T1.2 (Dr. Robin Anderson, Peter MacCallum Cancer Institute), SCP28(Dr. Joan Massague, Memorial Sloan Kettering Cancer Center), and AT3(Dr. Scott Abrams, Roswell Park Cancer Center).
Authentication	Cell lines either directly from ATCC or from original author.
Mycoplasma contamination	Cells regularly tested for mycoplasma.
Commonly misidentified lines (See ICLAC register)	N/A

Animals and other organisms

Policy information about studies involving animals; ARRIVE guidelines recommended for reporting animal research

Laboratory animals	C57B/6, NRG, athymic nude mice, Sp7-tTA,tetO-EGFP/cre (OSX-GFP) mice, B6.Cg-Tg(Cspg4-cre/Esr1*)BAkik/J (NG2-CreER), and B6.Cg-Gt(ROSA)26Sortm14(CAG-tdTomato)Hze/J (Ai14D) mice were purchased from Jackson Laboratory. NG2-RFP mice were generated by crossing NG2-CreER and Ai14D mice. The strain and number of mice used for each experiment are mentioned in text and legends.
Wild animals	N/A
Field-collected samples	N/A
Ethics oversight	All animal work was done in accordance with a protocol approved by the Baylor College of Medicine Institutional Animal Care and Use Committee. The investigator was not blinded to the group allocation during the whole experiment.

Note that full information on the approval of the study protocol must also be provided in the manuscript.

Flow Cytometry

Plots

Confirm that:

☒ The axis labels state the marker and fluorochrome used (e.g. CD4-FITC).

☒ The axis scales are clearly visible. Include numbers along axes only for bottom left plot of group (a 'group' is an analysis of identical markers).

☒ All plots are contour plots with outliers or pseudocolor plots.

☒ A numerical value for number of cells or percentage (with statistics) is provided.

Methodology

Sample preparation	Simple trypsinization, dilution
Instrument	BD FACS Aria II
Software	FloJo
Cell population abundance	See attached
Gating strategy	See attached

☒ Tick this box to confirm that a figure exemplifying the gating strategy is provided in the Supplementary Information.
